# Influence of clinical and neurocognitive factors in psychosocial functioning after a first episode non-affective psychosis: differences between males and females

**DOI:** 10.3389/fpsyt.2022.982583

**Published:** 2022-10-20

**Authors:** Maria Serra-Navarro, Silvia Amoretti, Norma Verdolini, María Florencia Forte, Ana M. Sánchez-Torres, Eduard Vieta, Derek Clougher, Antonio Lobo, Ana González-Pinto, Rocío Panadero, Alexandra Roldán, André F. Carvalho, Elena de la Serna, Alba Toll, J. A. Ramos-Quiroga, Carla Torrent, Manuel J. Cuesta, Miguel Bernardo, PEPs Group, Jairo González-Díaz

**Affiliations:** Barcelona Clinic Schizophrenia Unit, Hospital Clinic of Barcelona, Neuroscience Institute, August Pi I Sunyer Biomedical Research Institute (IDIBAPS), University of Barcelona, Barcelona, Spain; UR Center for Mental Health - CERSAME, School of Medicine and Health Sciences, Universidad del Rosario, Bogota DC, Colombia; Clínica Nuestra Señora de la Paz, Bogota DC, Colombia; Barcelona Clinic Schizophrenia Unit, Hospital Clinic of Barcelona, Neuroscience Institute, August Pi I Sunyer Biomedical Research Institute (IDIBAPS), University of Barcelona, Barcelona, Spain; Biomedical Research Networking Center for Mental Health Network (CIBERSAM), Barcelona, Spain; Department of Child and Adolescent Psychiatry, Hospital General Universitario Gregorio Marañón, School of Medicine, Institute of Psychiatry and Mental Health, Universidad Complutense, IiSGM, Madrid, Spain; Biomedical Research Networking Center for Mental Health Network (CIBERSAM), Barcelona, Spain; Department of Psychiatry, Hospital de la Santa Creu i Sant Pau, Institut d’Investigació Biomèdica-Sant Pau (IIB-SANT PAU), Universitat Autònoma de Barcelona (UAB), Barcelona, Spain; Biomedical Research Networking Center for Mental Health Network (CIBERSAM), Barcelona, Spain; Araba University Hospital, Bioaraba Research Institute, Vitoria-Gasteiz, Spain; University of the Basque Country (UPV-EHU), Vitoria-Gasteiz, Spain; Biomedical Research Networking Center for Mental Health Network (CIBERSAM), Barcelona, Spain; Department of Medicine and Psychiatry, Instituto de Investigación Sanitaria Aragón (IIS Aragón), Zaragoza University, Zaragoza, Spain; Biomedical Research Networking Center for Mental Health Network (CIBERSAM), Barcelona, Spain; Neurobiology Unit, Program in Neurosciences and Interdisciplinary Research Structure for Biotechnology and Biomedicine (BIOTECMED), Universitat de València, Biomedical Research Institute INCLIVA, Valencia, Spain; Neurobiology Unit, Program in Neurosciences and Interdisciplinary Research Structure for Biotechnology and Biomedicine (BIOTECMED), Universitat de València, Biomedical Research Institute INCLIVA, Valencia, Spain; Neurobiology Unit, Program in Neurosciences and Interdisciplinary Research Structure for Biotechnology and Biomedicine (BIOTECMED), Universitat de València, Biomedical Research Institute INCLIVA, Valencia, Spain; CIBERSAM, IDIBAPS, Department of Medicine, University of Barcelona, Barcelona, Spain; CIBERSAM, IDIBAPS, Department of Medicine, University of Barcelona, Barcelona, Spain; Barcelona Clinic Schizophrenia Unit, Hospital Clinic of Barcelona, Neuroscience Institute, August Pi I Sunyer Biomedical Research Institute (IDIBAPS), University of Barcelona, Barcelona, Spain; Department of Psychiatry and Psychology, Institute of Neuroscience, Hospital Clinic de Barcelona, Barcelona, Spain; Biomedical Research Networking Center for Mental Health Network (CIBERSAM), Barcelona, Spain; Department of Child and Adolescent Psychiatry and Psychology, 2017SGR881, Hospital Clínic Universitari, Institut Clinic de Neurociències, Barcelona, Spain; Biomedical Research Networking Center for Mental Health Network (CIBERSAM), Barcelona, Spain; Bellvitge University Hospital, IDIBELL, Barcelona, Spain; Biomedical Research Networking Center for Mental Health Network (CIBERSAM), Barcelona, Spain; Servicio de Salud del Principado de Asturias (SESPA) Oviedo, Instituto de Investigación Sanitaria del Principado de Asturias (ISPA), Instituto Universitario de Neurociencias del Principado de Asturias (INEUROPA), Oviedo, Spain; Department of Psychology, Universidad de Oviedo, Oviedo, Spain; Biomedical Research Networking Center for Mental Health Network (CIBERSAM), Barcelona, Spain; Servicio de Salud del Principado de Asturias (SESPA) Oviedo, Instituto de Investigación Sanitaria del Principado de Asturias (ISPA), Instituto Universitario de Neurociencias del Principado de Asturias (INEUROPA), Oviedo, Spain; Department of Psychiatry, Universidad de Oviedo, Oviedo, Spain; Biomedical Research Networking Center for Mental Health Network (CIBERSAM), Barcelona, Spain; Cruces University Hospital, BioCruces Health Research Institute, University of the Basque Country (UPV/EHU), Vizcaya, Spain; Biomedical Research Networking Center for Mental Health Network (CIBERSAM), Barcelona, Spain; Instituto de Investigación Sanitaria Hospital 12 de Octubre (imas12), Madrid, Spain; CogPsy Group, Universidad Complutense de Madrid (UCM), Madrid, Spain; Instituto de Investigación Sanitaria Hospital 12 de Octubre (imas12), Madrid, Spain; Parc Sanitari Sant Joan de Déu, Teaching, Research & Innovation Unit, Institut de Recerca Sant Joan de Déu, Sant Boi de Llobregat; Biomedical Research Networking Center for Mental Health Network (CIBERSAM), Barcelona, Spain; Hospital Infanto-juvenil Sant Joan de Déu, Institut de Recerca Sant Joan de Déu, Esplugues de Llobregat, Barcelona; Biomedical Research Networking Center for Mental Health Network (CIBERSAM), Barcelona, Spain; FIDMAG Germanes Hospitalàries Research Foundation, Barcelona, Spain; Biomedical Research Networking Center for Mental Health Network (CIBERSAM), Barcelona, Spain; Department of Psychiatry, Hospital Universitario Ramón y Cajal, IRYCIS, Universidad de Alcalá, Madrid, Spain; Department of Psychiatry, Complejo Hospitalario de Navarra, Pamplona, Spain; IdiSNA, Navarra Institute for Health Research, Pamplona, Spain; Biomedical Research Networking Center for Mental Health Network (CIBERSAM), Barcelona, Spain; Department of Medicine, University of Valencia, Valencia, Spain.; ^1^Bipolar and Depressive Disorders Unit, Hospital Clinic of Barcelona, Institute of Neurosciences, IDIBAPS, University of Barcelona, Barcelona, Catalonia, Spain; ^2^Biomedical Research Networking Center for Mental Health Network (CIBERSAM), Barcelona, Spain; ^3^Barcelona Clinic Schizophrenia Unit, Hospital Clinic of Barcelona, Neuroscience Institute, August Pi I Sunyer Biomedical Research Institute (IDIBAPS), University of Barcelona, Barcelona, Spain; ^4^Psychiatric Genetics Unit, Group of Psychiatry, Mental Health and Addictions, Vall d’Hebron Research Institute (VHIR), Barcelona, Catalonia, Spain; ^5^Local Health Unit Umbria 1, Department of Mental Health, Mental Health Center of Perugia, Perugia, Italy; ^6^Department of Psychiatry, Complejo Hospitalario de Navarra, Pamplona, Spain; ^7^IdiSNA, Navarra Institute for Health Research, Pamplona, Spain; ^8^Department of Medicine and Psychiatry, Instituto de Investigación Sanitaria Aragón (IIS Aragón), Zaragoza University, Zaragoza, Spain; ^9^Araba University Hospital, Bioaraba Research Institute, Vitoria-Gasteiz, Spain; ^10^Department of Psychiatry, University of the Basque Country (UPV-EHU), Vitoria-Gasteiz, Spain; ^11^Department of Child and Adolescent Psychiatry, Hospital General Universitario Gregorio Marañón, School of Medicine, Institute of Psychiatry and Mental Health, Universidad Complutense, IiSGM, Madrid, Spain; ^12^Department of Psychiatry, Hospital de la Santa Creu i Sant Pau, Institut d’Investigació Biomèdica-Sant Pau (IIB-SANT PAU), Universitat Autònoma de Barcelona (UAB), Barcelona, Spain; ^13^Innovation in Mental and Physical Health and Clinical Treatment (IMPACT), School of Medicine, Barwon Health, The Institute for Mental and Physical Health and Clinical Translation, Deakin University, Geelong, VIC, Australia; ^14^Department of Child and Adolescent Psychiatry and Psychology, 2017SGR881, Institut Clínic de Neurociències, Hospital Clínic Universitari, CIBERSAM, Institut d’Investigacions Biomèdiques August Pi i Sunyer (IDIBAPS), Department of Medicine, University of Barcelona, Barcelona, Spain; ^15^Hospital del Mar Medical Research Institute (IMIM), Universitat Autònoma de Barcelona (UAB), Barcelona, Spain; ^16^Department of Psychiatry, Hospital Universitari Vall d’Hebron, Barcelona, Catalonia, Spain; ^17^Department of Psychiatry and Legal Medicine, Universitat Autònoma de Barcelona, Barcelona, Catalonia, Spain

**Keywords:** first episode non-affective psychosis, psychosocial functioning, sex differences, cognition, negative symptoms

## Abstract

**Background:**

Deficits in psychosocial functioning are present in the early stages of psychosis. Several factors, such as premorbid adjustment, neurocognitive performance, and cognitive reserve (CR), potentially influence functionality. Sex differences are observed in individuals with psychosis in multiple domains. Nonetheless, few studies have explored the predictive factors of poor functioning according to sex in first-episode psychosis (FEP). This study aimed to explore sex differences, examine changes, and identify predictors of functioning according to sex after onset.

**Materials and methods:**

The initial sample comprised 588 individuals. However, only adults with non-affective FEP (*n* = 247, 161 males and 86 females) and healthy controls (*n* = 224, 142 males and 82 females) were included. A comprehensive assessment including functional, neuropsychological, and clinical scales was performed at baseline and at 2-year follow-up. A linear regression model was used to determine the predictors of functioning at 2-year follow-up.

**Results:**

FEP improved their functionality at follow-up (67.4% of both males and females). In males, longer duration of untreated psychosis (β = 0.328, *p* = 0.003) and worse premorbid adjustment (β = 0.256, *p* = 0.023) were associated with impaired functioning at 2-year follow-up, while in females processing speed (β = 0.403, *p* = 0.003), executive function (β = 0.299, *p* = 0.020) and CR (β = −0.307, *p* = 0.012) were significantly associated with functioning.

**Conclusion:**

Our data indicate that predictors of functioning at 2-year follow-up in the FEP group differ according to sex. Therefore, treatment and preventative efforts may be adjusted taking sex into account. Males may benefit from functional remediation at early stages. Conversely, in females, early interventions centered on CR enhancement and cognitive rehabilitation may be recommended.

## Introduction

Psychosocial functioning deficits are present in the early stages of psychosis (1, 2). Impairments are commonly observed in daily life activities. Although symptomatic remission was considered the critical treatment goal for a long time, and was the main focus in previous studies rather than functional recovery, there is increasing interest in addressing functional impairment. In terms of functional and clinical improvement, a recent meta-analysis found that long-term remission rate after a first psychotic episode was 58% and the recovery rate was 38% (3). According to the literature, several factors influence functionality. Certain factors are modifiable, including duration of untreated psychosis (DUP), negative symptoms (4–6), family environment, neurocognitive performance and cognitive reserve (CR) (1, 7, 8) and ergo are of potential clinical importance for therapeutic interventions. CR is defined as the coping capacity of the adult brain for pathology that minimizes symptomatology (9). Recently, it has been studied in severe mental disorders and interventions are being developed to enhance it given its potential protective properties (10–12). However, younger age, poorer premorbid adjustment, and male sex, are non-modifiable and represent a challenge to treatment, given the non-modifiable and represent a challenge to treatment, given their potential impact on psychosocial functioning (13–16).

Specific sex differences in neuroanatomy, neurofunctionality, gender and hormonal steroids have been reported in schizophrenia (SZ) individuals (17–19). Men show more severe gross neuroanatomical abnormalities than females (20) and these differences have even been observed in individuals with high genetic risk for the development of SZ; males have more neuroanatomical alterations than females when compared to same sex controls (18). In relation to hormonal steroids, Li et al. (21) found that estrogen deficiency is highly related to severity. Moreover, Mendrek et al. (20) identified an association between hormonal status and performance in neurocognitive domains. Regarding gender, a study from Lewine et al. (22) showed that female participants, independent of sex and diagnosis, performed better than males on neurocognition, with the exception of attention and executive function.

To date, males with SZ present more cognitive impairments than females and differences are less pronounced in the early stages of illness (20). Although, sex differences have been extensively described in first-episode psychosis (FEP), mixed results have been found in neurocognitive performance. For example, research has found that females demonstrate better premorbid adjustment (14, 23–25) and neurocognitive performance (14, 23) than their male counterparts. Conversely, higher severity of negative symptoms and higher rates of substance use were more frequent in males (23). Moreover, lower educational level, earlier age at onset, more severe illness course, poorer insight, more hospitalizations, and longer DUP were more frequent in male subjects with psychosis (13, 15, 23, 25–27). Regarding cognitive factors, females demonstrated better social cognition and verbal memory than men (25, 27), whilst males in the FEP group showed better performance on visuospatial (27) and working memory tasks (13).

A limited number of studies have explored the predictive factors of poor psychosocial functioning according to sex in FEP. Mattsson et al. (24) examined sex differences in the prediction of long-term outcomes in a group of 81 male and 72 females with FEP, and found that lower educational attainment level was the primary predictor of unfavorable outcome in females and a low premorbid level of functioning in males. Nevertheless, a notable limitation of this study was the lack of cognitive assessment despite cognitive performance being recognized as a reliable predictor of unfavorable outcomes in individuals with psychosis. Moreover, Willhite et al. (28) investigated sex differences in individuals at ultra-high-risk for developing a psychotic disorder and found that negative symptoms mediated differences in functioning between male and female subjects with FEP. These findings suggest that sex-based symptom presentation and functional outcome may predate conversion to psychosis (28). Therefore, identifying specific predictors according to sex may have important clinical implications for offering more personalized and targeted treatment in FEP.

There are two potential hypotheses that could explain these sex differences: hormone status and sex chromosomes. The first hypothesis is supported by the two peaks of incidence in females and the finding that females with SZ have more severe symptoms during the lower estrogen phase of their menstrual cycle (21, 29). Moreover, a study by Kaneda and Ohmori (30) showed that estradiol levels, a form of estrogen, are associated with severity of negative symptoms in males and may be a biological marker. The second hypothesis is based on recent genetic studies, which show that x-chromosome instability is involved and may contribute to the development of psychosis (21, 31).

Therefore, although research in the field is expanding, there remains a limited number of studies exploring sex differences in FEP, particularly in terms of clinical, neurocognitive, and psychosocial functioning. The present study addresses this gap in the literature by exploring these outcomes, while also identifying predictors of functionality in males and females, following a first episode non-affective psychosis. Specifically, and based on the research to date, we propose the following hypotheses: (1) differences will be found between males and females in clinical and neurocognitive outcomes; (2) changes from baseline to 2-year follow-up will be observed; and (3) we expect negative symptoms, neurocognition, CR and DUP to be predictors of psychosocial functioning. Specific sex differences are not hypothesized and will be explored accordingly. Similarly, the aims of the study were: (1) to explore sex differences in clinical, psychosocial functioning and neurocognitive outcomes; (2) to examine changes in neurocognition and functionality from baseline to 2-year follow-up; and (3) to identify predictors of functionality in males and females after a first episode non-affective psychosis.

## Materials and methods

### Study design and population

The sample was obtained from the “Phenotype-genotype interaction. Application of a predictive model in first psychotic episodes” (PEPs Project) study (32, 33). This is a multicenter, naturalistic, and longitudinal project under the umbrella of the Spanish Research Network on Mental Health (CIBERSAM) (34). The background, rationale and study design have been previously presented (32, 33). All participants were evaluated at two different time points. Individuals with FEP completed the full test battery of sociodemographic, clinical, functional, and neurocognitive assessments at both baseline and two-year follow-up. The same evaluation was administered to HCs at both stages, however, for the clinical assessment only the SCID-I-II was assessed.

Initially, the sample was composed of 588 individuals: 335 participants with a FEP and 253 healthy controls (HC). Inclusion criteria were: (1) aged between 7 and 35 years at the time of first evaluation; (2) duration of psychotic symptoms ≤1 year; (3) signed informed consent and (4) ability to speak Spanish. Exclusion criteria were as follows: (1) intellectual deficit according to DSM-IV criteria (including not only an IQ below 70 but also impaired functioning); (2) history of head injury with loss of consciousness and (3) organic disease with mental impact.

To ensure sample homogeneity, only adults with non-affective FEP and HCs were included. At two-year follow-up and according to DSM-IV-TR, non-affective FEP diagnosis was considered as: schizophrenia, schizophreniform, schizoaffective disorders, and other psychoses not otherwise specified. Supplementary Figure 1 shows the flowchart for the selection of the 247 non-affective FEP at baseline.

The FEP group was matched by age (±10%), gender and parental socioeconomic status (±1 level). The exclusion criteria for HC were the same as the FEP group, but also included the presence of a current or past psychotic disorder, major depression or other psychiatric illness and having a first-degree relative with psychotic disorder history. In this study, 224 adult HC were included.

Ethical approval was granted by the Hospital Clinic Ethics and Research Board. The study followed the ethical principles of the Declaration of Helsinki and Good Clinical Practices. All participants provided written informed consent.

### Assessments

#### Clinical and sociodemographic assessment

Sociodemographic and clinical data from all participants were collected at baseline and at two years follow-up. Hollingshead’s Two-Factor Index of Social Position (35) was used to define parental socioeconomic status (SES); Duration of Untreated Psychosis (DUP) was estimated considering the number of days between the presence of the first psychotic symptoms and the beginning of adequate treatment, and antipsychotic mean doses were calculated by chlorpromazine equivalents (CPZ) based on international consensus (36). Drug misuse habits were gathered using an adapted version of the European Adaptation of a Multidimensional Assessment Instrument for Drug and Alcohol Dependence scale (37). The Structured Clinical Interview for DSM (SCID-I-II) was used to establish diagnoses according to DSM-IV criteria (38, 39) in the FEP group. Conversely, the SCID-I-II was administered to assess HC’s mental health to ensure that they met the inclusion criteria and to rule out the exclusion criteria of the study.

In the FEP group, psychotic symptoms were assessed with the Positive and Negative Syndrome Scale (PANSS) (40), and affective symptoms with the Montgomery-Asberg Depression Rating Scale (MADRS) (41) and the Young Mania Rating Scale (YMRS) (42). A total score was obtained from each scale. Higher scores correspond to greater severity.

#### Functional assessment

The Functioning Assessment Short Test (FAST) was used to evaluate functional outcome (43, 44) from all participants. Higher scores represent higher disability.

#### Neurocognitive assessment

A neurocognitive battery was used to evaluate different cognitive domains through standardized instruments. The neuropsychological assessment was made in the second month of evaluation in order to ensure the psychopathologic stability of individuals with FEP and was repeated in the two-year follow-up visit. The neurocognitive battery measured the following cognitive domains: (1) Sustained attention was tested with the Continuous Performance Test–II (CPT-II) (45), version 5; (2) Verbal learning and memory, assessed with the Verbal Learning Test Spain Complutense for adults (TAVEC) (46); (3) Working memory was assessed by the Digit Span Subtest and the Letter-Number Sequencing Subtest of the Wechsler Adult Intelligence Scale (WAIS-III) (47); (4) Processing speed was assessed with the Trail making test (Form A) (TMT-A) (48); (5) The executive functions were evaluated using the Wisconsin Card Sorting Test (49), corrected by age and educational level; (6) Verbal fluency was evaluated using semantic fluency (animals) (50) and F-A-S tests (51); and (7) Managing Emotions was assessed with the Mayer-Salovey-Caruso Emotional Intelligence Test (MSCEIT) (52). All neurocognitive measures were transformed into T-scores. Higher scores correspond to better performance in all neurocognitive domains except for attention.

#### Cognitive reserve and premorbid adjustment assessment

Premorbid adjustment, namely levels of functioning before the onset of psychosis, was assessed with The Premorbid Adjustment Scale (PAS) (53). Only childhood and early adolescence life periods have been taken into account since they are the two periods answered by all the participants. Higher scores indicate worse premorbid adjustment.

Cognitive reserve (CR) was assessed using the three proposed proxy indicators of CR in FEP as described in previous literature (10, 54–56). Proxy indicators include education, estimated premorbid IQ, leisure, social, and physical activities. Education was measured with: patient’s completed years of study, performance during school, and parents’ educational level. Estimated premorbid IQ and crystallized intelligence were evaluated with the vocabulary subtest of WAIS-III which appears to remain stable during disease progression (57). A “cognitive reserve score” was created using a Principal Components Analysis (PCA) for each subject. Higher scores correspond to better performance for each subject. Higher scores correspond to better performance.

### Statistical analysis

Neurocognitive variables were grouped using PCA. The resulting cognitive domains were: sustained attention, verbal learning and memory, working memory, processing speed, executive functions, fluency, and emotion management (see Supplementary Table 1). Firstly, a multivariate analysis of variance with two factors: (1) sex (female vs. male) and (2) group (patients vs. HC) was conducted for demographic variables and for cognitive functions. For cognitive functions, a multivariate analysis of covariance with age and chlorpromazine equivalent as covariates was performed because FEP females and males were statistically different in these variables. In a second step, repeated measures multivariate analysis of covariance was used to check the differences and changes in cognitive functions with three factors: (1) sex (female vs. male), (2) group (patients vs. HC), and (3) assessment (baseline and follow-up). In patients, change scores were calculated using the reliable change index (RCI). To check changes for psychosocial functioning, RCI was calculated following this formula: (T2 − T1)/SED, where T1 and T2 are the individual’s observed baseline and 2-years follow-up scores, and SED is the standard error of the difference. For cognitive measures, corrections for measurement error and practice effects were then calculated for each participant using the RCI, calculated as (T2 − T1) − (M2 − M1)/SED, where T1 and T2 are the individual’s observed baseline and 2-year follow-up scores, M1 and M2 are the control group mean baseline and follow-up scores, and SED is the standard error of difference. An RCI [alpha set to 0.10 (two-tailed)] greater than +1.645 is considered a significant change, while reliable decline occurs when values fall below −1.645 (58–60). In the third step, in individuals with FEP, partial correlations controlling for chlorpromazine equivalent at baseline were computed for the continuous variables. The association between binary variables and the FAST score at 2-year follow-up was examined using a *t*-test, controlling again for the chlorpromazine equivalent effects. Finally, variables that were significantly correlated with FAST (*p* < 0.05) were included in two linear regression models with backward elimination in both sexes. In this way, we explored factors that predict psychosocial functioning at baseline and at 2-year follow-up, according to sex.

Statistical Package for the Social Sciences (SPSS v26) was used to analyze data. All statistical tests were carried out two-tailed, with an alpha level of significance set at *p* < 0.05.

## Results

### Sociodemographic, clinical, functional and cognitive characteristics of the sample and sex differences

A total of 247 FEP subjects (161 males and 86 females) and 224 HC (142 males and 82 females) were enrolled in this study. At two-year follow-up 145 individuals with FEP and 152 HC were re-evaluated as 102 subjects with FEP and 72 HC participants had withdrawn from the study due to a loss of follow-up or refusal of re-evaluation. The follow-up sample of the FEP group (*n* = 145) did not differ from the baseline sample in terms of sociodemographic, clinical, functional, and neurocognitive performance. Similarly, HCs assessed at follow-up (*n* = 152) showed no differences to the total sample of baseline HCs in terms of sociodemographic, functional, and the majority of cognitive domains.

A summary of the baseline sociodemographic and clinical characteristics of individuals with FEP and HC is shown in Table 1. There were main effects of group for SES (χ2 = 23.165, *p* < 0.001), tobacco and cannabis use (χ2 = 35.732, *p* < 0.001 and χ2 = 39.332, *p* < 0.001, respectively), psychosocial functioning (*t* = 437.177, *p* < 0.001), cognitive reserve (*F* = 155.857, *p* < 0.001), premorbid adjustment (*F* = 252.087, *p* < 0.001) and all cognitive measures. The male group was younger (*F* = 6.572, *p* = 0.011), reported higher tobacco and cannabis use (χ2 = 9.832, *p* = 0.007 and χ2 = 31.307, *p* < 0.001, respectively), higher CPZ (*F* = 5.720, *p* = 0.017), better attention (*F* = 11.466, *p* = 0.001) and processing speed (*F* = 12.640, *p* < 0.001), higher CR (*F* = 6.160, *p* = 0.013), and worse premorbid adjustment (*F* = 4.058, *p* = 0.045). No significant effect for GroupXSex was observed.

**TABLE 1:** Sex differences in sociodemographic, clinical and functional characteristics at baseline for subjects with psychosis and healthy controls.

	Subjects with psychosis (*n* = 247)		Healthy controls (*n* = 224)		Effect								
			
	Males (*n* = 161)	Females (*n* = 86)	Males (*n* = 142)	Females (*n* = 82)	Group			Sex			GroupXSex		
					F or χ2	p	ηp^2^ or Cramér’s V	F or χ2	p	ηp^2^ or Cramér’s V	F or χ2	p	ηp^2^
**Sociodemographic variables**													
Age (M ± SD)	24.96 ± 5.05	26.52 ± 5.31	25.58 ± 5.70	26.67 ± 5.50	0.542	0.462	0.001	6.572	**0.011**	0.014	0.204	0.652	0.000
SES, *N* (%)					23.165	**<0.001**	0.222	2.718	0.743	0.076			
High	29 (18)	14 (16)	30 (21)	20 (24)									
Medium-High	13 (8)	12 (14)	28 (20)	15 (18)									
Medium	45 (28)	16 (19)	40 (28)	22 (27)									
Medium-Low	52 (32)	27 (31)	37 (26)	21 (26)									
Low	18 (11)	15 (17)	6 (4)	3 (4)									
Missing value	4 (3)	2 (2)	1 (1)	1 (1)									
Tobacco use: Yes *N* (%)	120 (75)	50 (58)	63 (44)	30 (37)	35.732	**<0.001**	0.275	9.832	**0.007**	0.144			
Cannabis use: Yes *N* (%)	91 (57)	22 (26)	35 (25)	7 (9)	39.332	**<0.001**	0.289	31.307	**<0.001**	0.258			
**Clinical and functional variables** (M ± SD)													
PANSS positive	18.48 ± 8.01	18.79 ± 7.66	–	–	–	–	–	0.082	0.774	0.000	–	–	–
PANSS negative	19.53 ± 7.16	18.35 ± 8.80	–	–	–	–	–	1.288	0.257	0.005	–	–	–
PANSS general	38.06 ± 11.50	37.06 ± 12.34	–	–	–	–	–	0.402	0.527	0.002	–	–	–
PANSS total	76.08 ± 21.71	74.20 ± 24.77	–	–	–	–	–	0.378	0.539	0.002	–	–	–
MADRS score	12.01 ± 8.52	13.62 ± 9.72	–	–	–	–	–	1.802	0.181	0.007	–	–	–
YMRS score	8.22 ± 9.67	9.28 ± 10.30	–	–	–	–	–	0.637	0.425	0.003	–	–	–
DUP	104.85 ± 114.94	104.15 ± 136.91	–	–	–	–	–	0.002	0.968	0.000	–	–	–
CPZ	666.26 ± 500.82	486.57 ± 354.59	–	–	–	–	–	5.720	**0.017**	0.012	–	–	–
FAST	28.35 ± 15.64	30.71 ± 16.62	3.26 ± 8.92	3.10 ± 5.60	437.177	< **0.001**	0.491	0.769	0.381	0.002	0.994	0.319	0.002
**Cognitive measures** (M ± SD)													
Attention	179.09 ± 42.58	191.47 ± 47.77	145.26 ± 22.36	158.53 ± 29.97	14.255	< **0.001**	0.039	11.466	**0.001**	0.031	0.001	0.975	0.000
Verbal memory	198.97 ± 71.93	209.58 ± 72.73	285.41 ± 44.92	284.79 ± 50.17	48.118	< **0.001**	0.108	0.006	0.937	0.000	0.097	0.756	0.000
Working memory	73.92 ± 14.51	69.26 ± 13.77	88.78 ± 12.99	88.82 ± 42.26	21.361	< **0.001**	0.050	3.037	0.082	0.007	2.450	0.118	0.006
Processing speed	47.77 ± 11.43	42.78 ± 11.03	57.32 ± 8.85	55.41 ± 10.44	47.330	< **0.001**	0.103	12.640	< **0.001**	0.030	3.217	0.074	0.008
Executive function	200.81 ± 55.54	196.15 ± 68.07	231.80 ± 37.87	224.81 ± 47.01	6.766	**0.010**	0.018	0.617	0.433	0.002	0.082	0.774	0.000
Fluency	60.30 ± 11.35	60.28 ± 12.78	77.97 ± 13.66	75.73 ± 13.49	66.683	< **0.001**	0.145	1.686	0.195	0.004	0.243	0.622	0.001
Managing Emotions	259.83 ± 34.90	255.84 ± 28.04	283.87 ± 31.42	288.70 ± 35.21	33.350	< **0.001**	0.078	0.070	0.792	0.000	2.101	0.148	0.005
**Cognitive reserve and premorbid adjustment** (M ± SD)													
CR	75.12 ± 11.00	71.90 ± 12.28	88.85 ± 10.62	86.44 ± 10.85	155.857	< **0.001**	0.270	6.160	**0.013**	0.014	0.128	0.721	0.000
PAS	48.96 ± 24.57	43.47 ± 23.41	17.20 ± 12.06	15.04 ± 9.18	252.087	< **0.001**	0.362	4.058	**0.045**	0.009	0.769	0.381	0.002

M, Mean; SES, Socioeconomic status; PANSS, Positive and Negative Symptom Scale; MADRS, Montgomery-Asberg Depression Rating Scale; YMRS, Young Mania Rating Scale; DUP, Duration of Untreated Psychosis; CPZ, Chlorpromazine equivalents; FAST, Functioning Assessment Short Test; CR, Cognitive Reserve; PAS, Premorbid Adjustment Scale. Significant differences (*p* < 0.05) marked in bold.

At two-year follow-up, there were main effects of group for tobacco use, psychosocial functioning and all cognitive measures. The female group presented worse attention (*F* = 24.176, *p* < 0.001) and processing speed (*F* = 8.402, *p* = 0.004). Finally, there was a group x sex interaction for attention (*F* = 10.028, *p* = 0.002) (for more details see Supplementary Table 2). Supplementary Figure 2 shows the mean psychosocial functioning and cognitive scores with error bars in males and females with psychosis at baseline and follow-up.

### Changes in cognitive and clinical characteristics and psychosocial functioning

Results from the repeated measures ANOVA revealed a time effect for psychosocial functioning (*p* < 0.001, ηp^2^ = 0.065) and all cognitive domains (values of partial eta squared range from 0.021 to 0.144) except attention (*p* = 0.097), indicating an improvement for both groups from baseline to the 2-year follow-up. An improvement in clinical status was also observed (values of partial eta squared range from 0.200 to 0.396). There was a significant time x group effect observed only for psychosocial functioning (*p* < 0.001, ηp^2^ = 0.085), indicating a significantly different effect in the FEP group over time compared with the HC group. No significant effect for TimeXGroupXSex was observed (see Table 2).

**TABLE 2:** Changes in clinical, cognitive functions and psychosocial functioning.

	Time			Time × Group			Time × Sex			Time × Group × Sex		
	F	p	η p^2^	F	p	ηp^2^	F	p	ηp^2^	F	p	ηp^2^
**Clinical and functional variables** (M ± SD)												
PANSS positive	88.649	**<0.001**	0.384	–	–	–	0.289	0.592	0.002	–	–	–
PANSS negative	35.570	**<0.001**	0.200	–	–	–	0.067	0.796	0.000	–	–	–
PANSS general	84.817	**<0.001**	0.374	–	–	–	0.021	0.885	0.000	–	–	–
PANSS total	93.167	**<0.001**	0.396	–	–	–	0.031	0.860	0.000	–	–	–
MADRS score	42.766	**<0.001**	0.231	–	–	–	0.630	0.429	0.004	–	–	–
YMRS score	36.806	**<0.001**	0.205	–	–	–	0.328	0.568	0.002	–	–	–
FAST	19.709	**<0.001**	0.065	26.441	**<0.001**	0.085	0.024	0.876	0.000	0.773	0.380	0.003
**Cognitive measures** (M ± SD)												
Attention	2.775	0.097	0.013	0.444	0.506	0.002	1.044	0.308	0.005	3.131	0.078	0.014
Verbal memory	26.526	**<0.001**	0.096	0.975	0.324	0.004	0.350	0.555	0.001	0.001	0.980	0.000
Working memory	5.485	**0.020**	0.021	0.091	0.763	0.000	1.220	0.270	0.005	0.496	0.482	0.002
Processing speed	21.670	**<0.001**	0.077	3.172	0.076	0.012	0.031	0.860	0.000	1.810	0.180	0.007
Executive function	38.402	**<0.001**	0.144	0.416	0.520	0.002	1.120	0.291	0.005	0.779	0.379	0.003
Fluency	15.930	**<0.001**	0.062	0.529	0.468	0.002	0.031	0.861	0.000	0.130	0.718	0.001
Managing Emotions	8.817	**0.003**	0.037	0.156	0.694	0.001	0.013	0.908	0.000	0.728	0.394	0.003

PANSS, Positive and Negative Symptom Scale; MADRS, Montgomery-Asberg Depression Rating Scale; YMRS, Young Mania Rating Scale; FAST, Functioning Assessment Short Test. Significant differences (*p* < 0.05) marked in bold.

Supplementary Table 3 displays the percentage rates of improvements, declines and stability on all cognitive domains and psychosocial functioning as determined by the RCI. Most of the FEP improved their functionality on follow-up (67.4% of both males and females). Results herein indicate that although working memory improved over time, females improved less than males (*p* < 0.001, Cramer’s V = 0.403). No other significant group differences were observed.

### Predictors of psychosocial functioning

At baseline, in males with FEP, worse psychosocial functioning correlated with higher positive (*r* = 0.308, *p* < 0.001), negative (*r* = 0.234, *p* = 0.004), depressive (*r* = 0.287, *p* < 0.001) and manic symptoms (*r* = 0.231, *p* = 0.005), poorer performance in processing speed (*r* = –0.183, *p* = 0.035) and social cognition (*r* = –0.213, *p* = 0.017), and worse premorbid adjustment (*r* = 0.228, *p* = 0.006). In females, FAST was also correlated with positive (*r* = 0.467, *p* < 0.001), negative (*r* = 0.613, *p* < 0.001), depressive (*r* = 0.358, *p* = 0.001), manic symptoms (*r* = 0.272, *p* = 0.017), and premorbid adjustment (*r* = 0.433, *p* < 0.001). However, regarding neurocognitive performance, a significant correlation was observed in sustained attention only (*r* = 0.299, *p* = 0.025). For HCs, FAST correlated with premorbid adjustment (*r* = 0.324, *p* < 0.001) in males and in verbal memory (*r* = –0.345, *p* = 0.003), verbal fluency (*r* = –0.281, *p* = 0.018), and premorbid adjustment (*r* = 0.426, *p* < 0.001) in females. No other variables correlated with FAST at baseline (see Supplementary Table 4). At follow-up, worse psychosocial functioning in FEP males correlated with higher negative symptoms at baseline (*r* = 0.295, *p* = 0.003), longer DUP (*r* = 0.272, *p* = 0.010), higher CPZ (*r* = 0.295, *p* = 0.003), worse sustained attention (*r* = 0.346, *p* = 0.003), and worse premorbid adjustment (*r* = 0.359, *p* < 0.001). In FEP females, FAST was correlated with worse performance in processing speed (*r* = –0.424, *p* = 0.005), executive function (*r* = –0.350, *p* = 0.029) and lower cognitive reserve (r = –0.312, p = 0.032). In HCs none of the baseline variables were associated with psychosocial functioning at follow-up (Table 3).

**TABLE 3:** Correlations between psychosocial functioning (FAST) at follow-up and socio-demographic and clinical variables at baseline in subjects with psychosis and healthy controls.

	Subjects with psychosis (*n* = 145)				Healthy controls (*n* = 152)			
	Males (*n* = 97)		Females (*n* = 48)		Males (*n* = 101)		Females (*n* = 51)	
	Pearson Correlation, Student t, X^2^	*p*	Pearson Correlation, Student t, X^2^	*p*	Pearson Correlation, Student t, X^2^	*p*	Pearson Correlation, Student t, X^2^	*p*
**Sociodemographic variables**								
Age (M ± SD)	–0.016	0.874	–0.200	0.188	–0.092	0.366	–0.064	0.660
SES (%)	1.635	0.159	0.435	0.782	0.562	0.691	0.671	0.616
Tobacco use: Yes *N* (%)	1.379	0.171	0.218	0.828	0.444	0.658	1.137	0.261
Cannabis use: Yes *N* (%)	1.739	0.085	0.228	0.821	–0.472	0.638	–0.054	0.957
**Clinical and functional variables** (M ± SD)								
PANSS positive	0.150	0.145	0.080	0.600	–	–	–	–
PANSS negative	0.295	**0.003**	–0.029	0.852	–	–	–	–
PANSS general	0.271	**0.008**	0.099	0.519	–	–	–	–
PANSS total	0.289	**0.004**	0.067	0.662	–	–	–	–
MADRS score	0.166	0.107	0.226	0.135	–	–	–	–
YMRS score	0.023	0.822	0.085	0.579				
DUP	0.272	**0.010**	0.063	0.690	–	–	–	–
**Cognitive measures** (M ± SD)								
Attention	0.346	**0.003**	0.261	0.129	0.009	0.932	0.088	0.549
Verbal memory	–0.192	0.074	–0.181	0.264	0.035	0.739	0.180	0.220
Working memory	–0.177	0.095	–0.031	0.844	–0.137	0.180	–0.062	0.674
Processing speed	–0.101	0.342	–0.424	**0.005**	0.018	0.863	–0.068	0.644
Executive function	–0.025	0.820	–0.350	**0.029**	0.110	0.298	–0.270	0.067
Fluency	–0.064	0.559	–0.100	0.538	0.131	0.207	–0.181	0.223
Managing Emotions	–0.118	0.281	–0.013	0.937	–0.074	0.477	0.056	0.706
**Cognitive reserve and premorbid adjustment** (M ± SD)								
CR	–0.135	0.206	–0.312	**0.032**	–0.046	0.654	0.039	0.795
PAS	0.359	< **0.001**	0.268	0.090	0.103	0.314	0.039	0.795

M, Mean; SES, Socioeconomic status; PANSS, Positive and Negative Symptom Scale; MADRS, Montgomery-Asberg Depression Rating Scale; YMRS, Young Mania Rating Scale; DUP, Duration of Untreated Psychosis; CPZ, Chlorpromazine equivalents; FAST, Functioning Assessment Short Test; CR, Cognitive Reserve; PAS, Premorbid Adjustment Scale. Significant differences (*p* < 0.05) marked in bold.

Results for linear regression are reported in Table 4. At baseline in FEP males, after including the variables that reached statistical significance in bivariate analyses in the regression model and controlling for chlorpromazine equivalent, positive (β = 0.285, *p* < 0.001), depressive symptoms (β = 0.195, *p* = 0.014) and adjustment (β = 0.213, *p* = 0.006) were significantly associated with worse FAST, with a higher effect exerted by positive symptoms. At follow-up, longer DUP (β = 0.328, *p* = 0.003) and worse premorbid adjustment (β = 0.256, *p* = 0.023) were associated with impaired psychosocial functioning.

**TABLE 4:** Linear regression of the socio-demographic, clinical and neuropsychological variables associated with psychosocial functioning in males and females with psychosis.

		Model	ßeta	*t*	*p*
Males	Baseline	*R* = 0.452, *R*^2^ = 0.504, *F* = 12.045, df (3), *p* < 0.001			
		PANSS positive	0.285	3.684	**<0.001**
		MADRS score	0.195	2.499	**0.014**
		Premorbid adjustment	0.213	2.803	**0.006**
		Constant		2.149	0.033
	Follow-up	*R* = 0.619, *R*^2^ = 0.383, *F* = 12.227, df (2), *p* <0.001			
		DUP	0.328	3.044	**0.003**
		Premorbid adjustment	0.256	2.342	**0.023**
		Constant		1.150	0.255
Females	Baseline	*R* = 0.770, *R*^2^ = 0.592, *F* = 17.063, df (4), *p* <0.001			
		PANSS negative	0.655	6.553	<**0.001**
		YMRS score	0.412	4.387	<**0.001**
		Sustained attention	0.248	2.599	**0.012**
		Constant		–1.054	0.297
	Follow-up	*R* = 0.692, *R*^2^ = 0.479, *F* = 8.030, df (3), *p* < 0.001			
		Processing speed	–0.403	3.142	**0.003**
		Executive function	–0.299	2.434	**0.020**
		Cognitive reserve	–0.307	–2.153	**0.038**
		Constant		0.655	0.516

Significant differences (*p* < 0.05) are bold.

In females, at baseline, negative (β = 0.655, *p* < 0.001), manic symptoms (β = 0.412, *p* < 0.001) and worse attention performance (β = 0.248, *p* = 0.012) were significantly associated with worse functioning, while at follow-up processing speed (β = –0.403, *p* = 0.003), executive function (β = –0.299, *p* = 0.020) and CR (β = –0.307, *p* = 0.02) were significantly associated with FAST, with a higher effect exerted by processing speed.

## Discussion

Two main findings emerged from the present study. Firstly, individuals with FEP had higher substance abuse, lower SES, worse psychosocial functioning and neurocognitive performance in all cognitive domains in relation to the HC group (27, 61). As expected, individuals with FEP present more difficulties and impairments than the HC group. Secondly, different effects of sex were found regardless of the group. The male group uses more tobacco and cannabis, are younger, have higher CR and CPZ doses, worse premorbid adjustment and perform better in attention and processing speed. These differences have been found in individuals with schizophrenia. Males tend to show a higher incidence of the disorder, an earlier age at onset, poorer premorbid adjustment, higher rates of substance abuse, worse psychosocial functioning, and a more severe course of the disease, especially in negative symptoms, while in the FEP group mixed results have been found (14, 62, 63). In addition, males with psychosis require higher doses of antipsychotic than females (21). In terms of premorbid adjustment, no significant differences were found in our study, failing to replicate the results of Cotton et al. (64). Previous literature suggests that males have greater negative symptoms than females with FEP, especially related to emotional withdrawal, blunted affects, and avolition-apathy (13, 25, 65, 66). However, in our study, no significant differences were found in this regard. These results might be interpreted in the light of different models that explain SZ spectrum disorders. The neurodevelopmental model of SZ posits that the illness is the end stage of abnormal neurodevelopmental processes that began years before the onset of the illness (64). Within this theoretical framework, an attempt to describe relationships between sex/gender and indicators of neurodevelopment compromise in SZ has been made, but no associations regarding sex were found (62).

There are some possible explanations for the inconsistency in results. First, no specific scale was used to assess negative symptoms as the PANSS was the chosen measurement in this study. Similar to our results, González-Rodríguez et al. (65) found no significant gender differences in psychopathology assessed by the PANSS in FEP. Secondly, Willhite et al. (28) found no differences at baseline in negative symptoms when they studied gender differences in a simple of high-risk individuals. Regarding processing speed, it has been shown that females with schizophrenia have poorer processing speed when evaluated using the TMT-A (67). However, these findings were not replicated by Zanelli et al. (68) who found no sex differences in neurocognition among FEP. The female group showed worse sustained attention performance than males. Research has shown differences between sex, for example, females showed slower reaction times (69) and were less aroused than males (70). Similar results were described by Hsieh et al. (71) who evaluated sustained attention with the CPT in a sample of 900 adults and found that males outperformed females. Thus, sex differences in neurocognitive performance are a controversial issue as results remain inconclusive. The heterogeneity in the results may be partly explained by differences in the studied populations, namely FEP or schizophrenia, as well as the subtest used to measure each domain. To compare the results and generalize the findings, future research should endeavor to homogenize evaluation tools to assess the neurocognitive domains and the stage of the illness.

Regarding changes, both groups improved at follow-up and no differences were found between males and females. According to previous literature, FEP individuals improve in functional outcomes, achieved recovery and demonstrated symptom remission (54, 72). With reference to gender, we found that men improved significantly more than women in working memory. To the best of our knowledge, this has not yet been documented in the current literature.

Although no clinical or psychosocial functioning differences have been found between sexes, it seems that the variables associated with poorer functioning differ according to sex. For the prediction of psychosocial functioning, worse premorbid adjustment and higher were predictors of worse psychosocial functioning in males. The relationship between premorbid adjustment and DUP with psychosocial functioning have been widely reported (73). Premorbid adjustment is also considered a predictor of clinical severity, especially for subjects with FEP presenting negative symptoms (5, 10).

In females, worse processing speed, worse executive function and lower CR level predicted worse psychosocial functioning. In severe mental illness, CR has a significant influence on cognitive, clinical and functional outcomes (11, 74). In FEP, individuals with CR perform better in neurocognitive scales and functioning (1, 10, 54). A lower educational level predicted worse results in females but not in males (24). Regarding processing speed, Lindgren et al. (75), reported that it was associated with 1-year remission, occupational status, and maintaining of life goals and Milev et al. (76) found that in FEP, verbal memory, processing speed, and attention were potential targets for psychosocial interventions to improve outcome. In fact, it has been shown that processing speed is related to functioning in individuals with ultra-high-risk for psychosis (77). In line with our results, a recent meta-analysis found that there is a positive association between executive function and psychosocial function (78).

**FIGURE 1:**
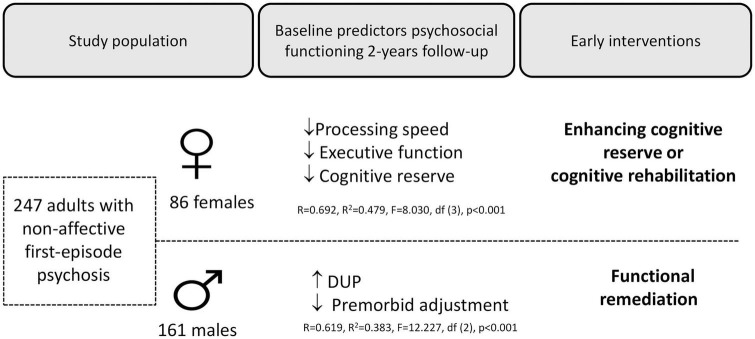
Overview of the study population, the main results, and the recommended intervention.

Thus, it seems that the predictors of psychosocial functioning at 2-year follow-up in the FEP group differ according to sex (see Figure 1). These sex differences could have important clinical implications, not only in terms of diagnosis, but also in terms of therapeutic approach. In the case of the former, and in order to improve psychosocial functioning, the present study supports the need to perform a thorough assessment of CR, premorbid adjustment, DUP, and neurocognitive status. In terms of therapeutic options, based on these results, we propose that males with FEP may benefit from a functional remediation from early stages. Conversely, in females, the implementation of early interventions centered on CR enhancement and cognitive rehabilitation may be beneficial, as CR has been associated with better cognitive performance and psychosocial functioning (1, 10, 54) and is a reliable predictor of functionality in this group.

This study has certain limitations which must be taken into account. Firstly, when participants were evaluated, there was no validated instrument to measure CR so criteria established and replicated in previous studies were followed. Secondly, no specific scale was used to assess negative symptomatology. Future studies making use of newer and improved negative symptom scales — such as the Brief Negative Symptom Scale (BNSS) (79) or the Clinical Assessment Interview for Negative Symptoms (CAINS) (80) — may be more appropriate for their evaluation. Thirdly, another limitation is the difference between the group size of males and females. In the female group, the small sample size may have interfered with the results (low statistical power). This is possibly due to the naturalistic and multicentric nature of the study with a representative sample of non-affective FEP in a stable clinical phase recruited from the whole Spanish territory. Thus, further studies with larger sample sizes are required to confirm these findings.

In conclusion, this study identified premorbid adjustment, and DUP as risk factors in males with FEP. Similarly, processing speed, executive function and CR were recognized as modifiable factors in females. These results suggest that the enhancement of modifiable factors may improve functional outcomes and could be beneficial in the development of early intervention programs. Future studies, using larger sample sizes are needed to determine which factors might influence the relationship between neurocognitive or clinical outcomes and functioning depending on sex. Finally, longitudinal studies would help to understand the long-term impact of these findings.

## Data availability statement

The raw data supporting the conclusions of this article will be made available by the authors, without undue reservation.

## Ethics statement

The studies involving human participants were reviewed and approved by the Hospital Ethics Committee (2008/4232), according to the Declaration of Helsinki principles and Good Clinical Practice guidelines, within the requirements of the Spanish Law and the State Regulatory Authority. The patients/participants provided their written informed consent to participate in this study.

## PEPs Group

Jairo González-Díaz: Barcelona Clinic Schizophrenia Unit, Hospital Clinic of Barcelona, Neuroscience Institute, August Pi I Sunyer Biomedical Research Institute (IDIBAPS), University of Barcelona, Barcelona, Spain; UR Center for Mental Health - CERSAME, School of Medicine and Health Sciences, Universidad del Rosario, Bogota DC, Colombia; Clínica Nuestra Señora de la Paz, Bogota DC, Colombia. Lucila Barbosa: Barcelona Clinic Schizophrenia Unit, Hospital Clinic of Barcelona, Neuroscience Institute, August Pi I Sunyer Biomedical Research Institute (IDIBAPS), University of Barcelona, Barcelona, Spain. Covadonga M. Diaz-Caneja and Marta Rapado-Castro: Biomedical Research Networking Center for Mental Health Network (CIBERSAM), Barcelona, Spain; Department of Child and Adolescent Psychiatry, Hospital General Universitario Gregorio Marañón, School of Medicine, Institute of Psychiatry and Mental Health, Universidad Complutense, IiSGM, Madrid, Spain. Carlo Alemany and Aina Avila-Parcet: Biomedical Research Networking Center for Mental Health Network (CIBERSAM), Barcelona, Spain; Department of Psychiatry, Hospital de la Santa Creu i Sant Pau, Institut d’Investigació Biomèdica-Sant Pau (IIB-SANT PAU), Universitat Autònoma de Barcelona (UAB), Barcelona, Spain. Iñaki Zorrilla and Itxasco Gonzalez-Ortega: Biomedical Research Networking Center for Mental Health Network (CIBERSAM), Barcelona, Spain; Araba University Hospital, Bioaraba Research Institute, Vitoria-Gasteiz, Spain; University of the Basque Country (UPV-EHU), Vitoria-Gasteiz, Spain. Concepción De-la-Cámara and Pedro Saz: Biomedical Research Networking Center for Mental Health Network (CIBERSAM), Barcelona, Spain; Department of Medicine and Psychiatry, Instituto de Investigación Sanitaria Aragón (IIS Aragón), Zaragoza University, Zaragoza, Spain. Juan Nacher: Biomedical Research Networking Center for Mental Health Network (CIBERSAM), Barcelona, Spain; Neurobiology Unit, Program in Neurosciences and Interdisciplinary Research Structure for Biotechnology and Biomedicine (BIOTECMED), Universitat de València, Biomedical Research Institute INCLIVA, Valencia, Spain. Esther Lorente: Neurobiology Unit, Program in Neurosciences and Interdisciplinary Research Structure for Biotechnology and Biomedicine (BIOTECMED), Universitat de València, Biomedical Research Institute INCLIVA, Valencia, Spain. Teresa Legido: CIBERSAM, IDIBAPS, Department of Medicine, University of Barcelona, Barcelona, Spain. Francesc Casanovas: CIBERSAM, IDIBAPS, Department of Medicine, University of Barcelona, Barcelona, Spain. Nestor Arbelo: Barcelona Clinic Schizophrenia Unit, Hospital Clinic of Barcelona, Neuroscience Institute, August Pi I Sunyer Biomedical Research Institute (IDIBAPS), University of Barcelona, Barcelona, Spain. Lidia Ilzarbe: Department of Psychiatry and Psychology, Institute of Neuroscience, Hospital Clinic de Barcelona, Barcelona, Spain. Josefina Castro-Fornieles: Biomedical Research Networking Center for Mental Health Network (CIBERSAM), Barcelona, Spain; Department of Child and Adolescent Psychiatry and Psychology, 2017SGR881, Hospital Clínic Universitari, Institut Clinic de Neurociències, Barcelona, Spain. Immaculada Baeza: Biomedical Research Networking Center for Mental Health Network (CIBERSAM), Barcelona, Spain; Department of Child and Adolescent Psychiatry and Psychology, 2017SGR881, Hospital Clínic Universitari, Institut Clinic de Neurociències, Barcelona, Spain. Fernando Contreras: Biomedical Research Networking Center for Mental Health Network (CIBERSAM), Barcelona, Spain; Bellvitge University Hospital, IDIBELL, Barcelona, Spain. Teresa Bobes Bascarán: Biomedical Research Networking Center for Mental Health Network (CIBERSAM), Barcelona, Spain; Servicio de Salud del Principado de Asturias (SESPA) Oviedo, Instituto de Investigación Sanitaria del Principado de Asturias (ISPA), Instituto Universitario de Neurociencias del Principado de Asturias (INEUROPA), Oviedo, Spain; Department of Psychology, Universidad de Oviedo, Oviedo, Spain. Leticia González-Blanco: Biomedical Research Networking Center for Mental Health Network (CIBERSAM), Barcelona, Spain; Servicio de Salud del Principado de Asturias (SESPA) Oviedo, Instituto de Investigación Sanitaria del Principado de Asturias (ISPA), Instituto Universitario de Neurociencias del Principado de Asturias (INEUROPA), Oviedo, Spain; Department of Psychiatry, Universidad de Oviedo, Oviedo, Spain. Rafael Segarra Echevarría and Arantzazu Zabala Rabadán: Biomedical Research Networking Center for Mental Health Network (CIBERSAM), Barcelona, Spain; Cruces University Hospital, BioCruces Health Research Institute, University of the Basque Country (UPV/EHU), Vizcaya, Spain. Roberto Rodriguez-Jimenez: Biomedical Research Networking Center for Mental Health Network (CIBERSAM), Barcelona, Spain; Instituto de Investigación Sanitaria Hospital 12 de Octubre (imas12), Madrid, Spain; CogPsy Group, Universidad Complutense de Madrid (UCM), Madrid, Spain. Luis Sanchez-Pastor: Instituto de Investigación Sanitaria Hospital 12 de Octubre (imas12), Madrid, Spain. Judith Usall: Parc Sanitari Sant Joan de Déu, Teaching, Research & Innovation Unit, Institut de Recerca Sant Joan de Déu, Sant Boi de Llobregat. Anna Butjosa: Biomedical Research Networking Center for Mental Health Network (CIBERSAM), Barcelona, Spain; Hospital Infanto-juvenil Sant Joan de Déu, Institut de Recerca Sant Joan de Déu, Esplugues de Llobregat, Barcelona. Salvador Sarró and María Ángeles García León: Biomedical Research Networking Center for Mental Health Network (CIBERSAM), Barcelona, Spain; FIDMAG Germanes Hospitalàries Research Foundation, Barcelona, Spain. Ángela Ibáñez: Biomedical Research Networking Center for Mental Health Network (CIBERSAM), Barcelona, Spain; Department of Psychiatry, Hospital Universitario Ramón y Cajal, IRYCIS, Universidad de Alcalá, Madrid, Spain. Lucía Moreno-Izco: Department of Psychiatry, Complejo Hospitalario de Navarra, Pamplona, Spain; IdiSNA, Navarra Institute for Health Research, Pamplona, Spain. Vicent Balanzá-Martinez: Biomedical Research Networking Center for Mental Health Network (CIBERSAM), Barcelona, Spain; Department of Medicine, University of Valencia, Valencia, Spain.

## Author contributions

SA and MS-N designed the study, managed the literature searches and analyses, undertook the statistical analysis, and wrote the first draft of the manuscript. CT and EV revised the first draft and added critical comments to guide the redaction of the final manuscript. NV, MF, AS-T, DC, AL, AG-P, RP, AR, AC, ES, AT, MC, and MB revised the second draft of the article and provided critical comments to guide the redaction of the final manuscript. All authors within the PEPs Group recruited individuals with FEP and healthy controls at their centers, provided the anonymous data and revise the final manuscript and approved the final manuscript.
