# Corrigendum: Influence of clinical and neurocognitive factors in psychosocial functioning after a first episode non-affective psychosis: differences between males and females

**DOI:** 10.3389/fpsyt.2023.1200313

**Published:** 2023-06-13

**Authors:** Maria Serra-Navarro, Silvia Amoretti, Norma Verdolini, María Florencia Forte, Ana M. Sánchez-Torres, Eduard Vieta, Derek Clougher, Antonio Lobo, Ana González-Pinto, Rocío Panadero, Alexandra Roldán, André F. Carvalho, Elena de la Serna, Alba Toll, J. A. Ramos-Quiroga, Carla Torrent, Manuel J. Cuesta, Miguel Bernardo, PEPs Group

**Affiliations:** ^1^Bipolar and Depressive Disorders Unit, Hospital Clinic of Barcelona, Institute of Neurosciences, IDIBAPS, University of Barcelona, Barcelona, Catalonia, Spain; ^2^Biomedical Research Networking Center for Mental Health Network (CIBERSAM), Barcelona, Spain; ^3^Barcelona Clinic Schizophrenia Unit, Hospital Clinic of Barcelona, Neuroscience Institute, August Pi I Sunyer Biomedical Research Institute (IDIBAPS), University of Barcelona, Barcelona, Spain; ^4^Psychiatric Genetics Unit, Group of Psychiatry, Mental Health and Addictions, Vall d'Hebron Research Institute (VHIR), Barcelona, Catalonia, Spain; ^5^Local Health Unit Umbria 1, Department of Mental Health, Mental Health Center of Perugia, Perugia, Italy; ^6^Department of Psychiatry, Complejo Hospitalario de Navarra, Pamplona, Spain; ^7^IdiSNA, Navarra Institute for Health Research, Pamplona, Spain; ^8^Department of Medicine and Psychiatry, Instituto de Investigación Sanitaria Aragón (IIS Aragón), Zaragoza University, Zaragoza, Spain; ^9^Araba University Hospital, Bioaraba Research Institute, Vitoria-Gasteiz, Spain; ^10^Department of Psychiatry, University of the Basque Country (UPV-EHU), Vitoria-Gasteiz, Spain; ^11^Department of Child and Adolescent Psychiatry, Hospital General Universitario Gregorio Marañón, School of Medicine, Institute of Psychiatry and Mental Health, Universidad Complutense, IiSGM, Madrid, Spain; ^12^Department of Psychiatry, Hospital de la Santa Creu i Sant Pau, Institut d'Investigació Biomèdica-Sant Pau (IIB-SANT PAU), Universitat Autònoma de Barcelona (UAB), Barcelona, Spain; ^13^Innovation in Mental and Physical Health and Clinical Treatment (IMPACT), School of Medicine, Barwon Health, The Institute for Mental and Physical Health and Clinical Translation, Deakin University, Geelong, VIC, Australia; ^14^Department of Child and Adolescent Psychiatry and Psychology, 2017SGR881, Institut Clínic de Neurociències, Hospital Clínic Universitari, CIBERSAM, Institut d'Investigacions Biomèdiques August Pi i Sunyer (IDIBAPS), Department of Medicine, University of Barcelona, Barcelona, Spain; ^15^Hospital del Mar Medical Research Institute (IMIM), Universitat Autònoma de Barcelona (UAB), Barcelona, Spain; ^16^Department of Psychiatry, Hospital Universitari Vall d'Hebron, Barcelona, Catalonia, Spain; ^17^Department of Psychiatry and Legal Medicine, Universitat Autònoma de Barcelona, Barcelona, Catalonia, Spain

**Keywords:** first episode non-affective psychosis, psychosocial functioning, sex differences, cognition, negative symptoms

In the published article, there was an error. The title of a sub-section is incorrect.

A correction has been made to Results, Changes in cognitive functions and psychosocial functioning, paragraph 4. This title previously stated:

“Changes in cognitive functions and psychosocial functioning”

The corrected sentence appears below:

“Changes in cognitive and clinical characteristics and psychosocial functioning”

In the published article, there was an error. Part of a paragraph was incorrectly included.

A correction has been made to Discussion, paragraph 1. This sentence previously stated:

“Two main findings emerged from the present study. Firstly, individuals with FEP had higher substance abuse, lower SES, worse psychosocial functioning and neurocognitive performance in all cognitive domains in relation to the HC group (27, 61). As expected, individuals with FEP present more difficulties and impairments than the HC group. Secondly, different effects of sex were found regardless of the group. The male group uses more tobacco and cannabis, are younger, have higher CR and CPZ doses, worse premorbid adjustment and perform better in attention and processing speed. These differences have been found in individuals with schizophrenia. Males tend to show a higher incidence of the disorder, an earlier age at onset, poorer premorbid adjustment, higher rates of substance abuse, worse psychosocial functioning, and a more severe course of the disease, especially in negative symptoms, while in the FEP group mixed results have been found (14, 62). In addition, males with psychosis require higher doses of antipsychotic than females (21). In terms of premorbid adjustment, no significant differences were found in our study, failing to replicate the results of Cotton et al. (64). Previous literature suggests that males have greater negative symptoms than females with FEP, especially related to emotional withdrawal, blunted affects, and avolition-apathy (13, 25, 65, 66). However, in our study, no significant differences were found in this regard. These results might be interpreted in the light of different models that explain SZ spectrum disorders. The neurodevelopmental model of SZ posits that the illness is the end stage of abnormal neurodevelopmental processes that began years before the onset of the illness (64). Within this theoretical framework, an attempt to describe relationships between sex/gender and indicators of neurodevelopment compromise in SZ has been made, but no associations regarding sex were found (62). In SZ, sex differences have been found, were men tend to show a higher incidence of the disorder, an earlier age at onset, poorer premorbid adjustment, higher rates of substance abuse, worse psychosocial functioning, and a more severe course of the disease, especially in negative symptoms (14, 62, 66) while in the FEP group mixed results have been found. In fact, in the present study no differences were found in regards to clinical or functional aspects. This could be interpreted in light of the neurodegenerative hypothesis (64), suggesting that SZ is a disorder that debuts at an early age, and from that moment on, a neurodegenerative process begins and the subject progressively loses capacities.”

The corrected sentence appears below:

“Two main findings emerged from the present study. Firstly, individuals with FEP had higher substance abuse, lower SES, worse psychosocial functioning and neurocognitive performance in all cognitive domains in relation to the HC group (27, 61). As expected, individuals with FEP present more difficulties and impairments than the HC group. Secondly, different effects of sex were found regardless of the group. The male group uses more tobacco and cannabis, are younger, have higher CR and CPZ doses, worse premorbid adjustment and perform better in attention and processing speed. These differences have been found in individuals with schizophrenia. Males tend to show a higher incidence of the disorder, an earlier age at onset, poorer premorbid adjustment, higher rates of substance abuse, worse psychosocial functioning, and a more severe course of the disease, especially in negative symptoms, while in the FEP group mixed results have been found (14, 62, 63). In addition, males with psychosis require higher doses of antipsychotic than females (21). In terms of premorbid adjustment, no significant differences were found in our study, failing to replicate the results of Cotton et al. (64). Previous literature suggests that males have greater negative symptoms than females with FEP, especially related to emotional withdrawal, blunted affects, and avolition-apathy (13, 25, 65, 66). However, in our study, no significant differences were found in this regard. These results might be interpreted in the light of different models that explain SZ spectrum disorders. The neurodevelopmental model of SZ posits that the illness is the end stage of abnormal neurodevelopmental processes that began years before the onset of the illness (64). Within this theoretical framework, an attempt to describe relationships between sex/gender and indicators of neurodevelopment compromise in SZ has been made, but no associations regarding sex were found (62).”

In the published article, the citation 66, Choi et al., 2009 was incorrectly placed and numbered.

The reference should be cited in the sentence: “mixed results have been found (Ochoa et al., 2012; Navarra-Ventura et al., 2021; Choi et al., 2009) (14, 62, 63). In addition, males with psychosis require higher doses”, and be numbered as reference 63:

63. Choi JS, Chon MW, Kang DH, Jung MH, Kwon JS. Gender difference in the prodromal symptoms of first-episode Schizophrenia. *J Korean Med Sci*. (2009) 24:1083–8. doi: 10.3346/jkms.2009.24.6.1083

As consequence, references 63 (Cotton SM et al. 2009), 64 (González-Rodríguez A, 2014) and 65 (Hui CLM, 2016) were renumbered as:

64. Cotton SM, Lambert M, Schimmelmann BG, Foley DL, Morley KI, McGorry PD, et al. Gender differences in premorbid, entry, treatment, and outcome characteristics in a treated epidemiological sample of 661 patients with first episode psychosis. *Schizophr Res*. (2009) 114:17–24. doi: 10.1016/j.schres.2009.07.002

65. González-Rodríguez A, Studerus E, Spitz A, Bugra H, Aston J, Borgwardt S, et al. Gender Differences in the Psychopathology of Emerging Psychosis. *Isr J Psychiatry Relat Sci*. (2014) 51:85–92.

66. Hui CLM, Leung CM, Chang WC, Chan SKW, Lee EHM, Chen EYH. Examining gender difference in adult-onset psychosis in Hong Kong. *Early Interv Psychiatry*. (2016) 10:324–33. doi: 10.1111/eip.12167

The authors apologize for this error and state that this does not change the scientific conclusions of the article in any way. The original article has been updated.
