# Supplementary figures and images for: Influence of clinical and neurocognitive factors in psychosocial functioning after a first episode non-affective psychosis: differences between males and females
Source: Front Psychiatry. 2022 Oct 20;13:982583. doi: 10.3389/fpsyt.2022.982583 (PMC9632657; doi:10.3389/fpsyt.2022.982583)

**Supplementary Figure 1.** Flowchart for the selection of the 247 non-affective FEP at baseline

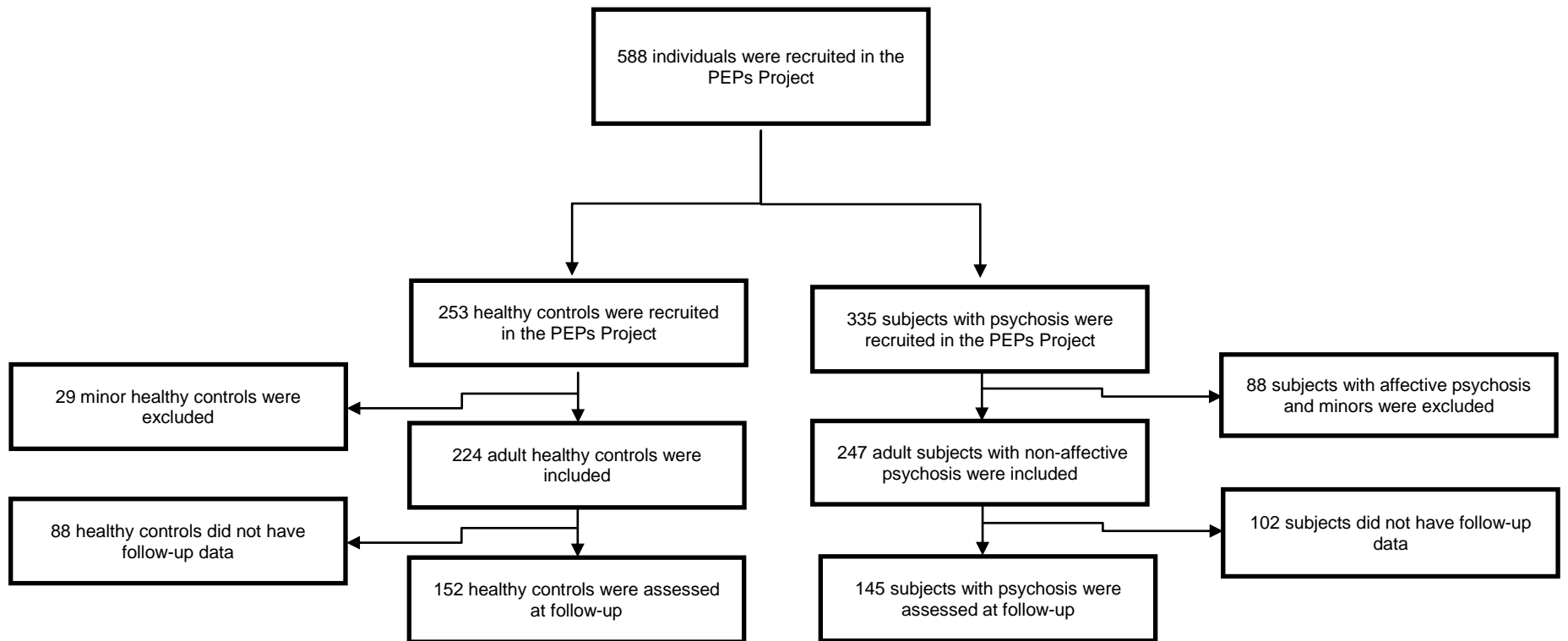

Supplement: Supplementary file 1 [file Data_Sheet_1.PDF]
