# Supplementary material for: Influence of clinical and neurocognitive factors in psychosocial functioning after a first episode non-affective psychosis: differences between males and females
Source: Front Psychiatry. 2022 Oct 20;13:982583. doi: 10.3389/fpsyt.2022.982583 (PMC9632657; doi:10.3389/fpsyt.2022.982583)
Supplement: Supplementary file 2 [file Data_Sheet_2.PDF]

Supplementary Figure 2. Mean psychosocial functioning and cognitive scores with error bars in males and females with psychosis at baseline and follow-up

Psychosocial functioning (Functioning Assessment Short Test, FAST)

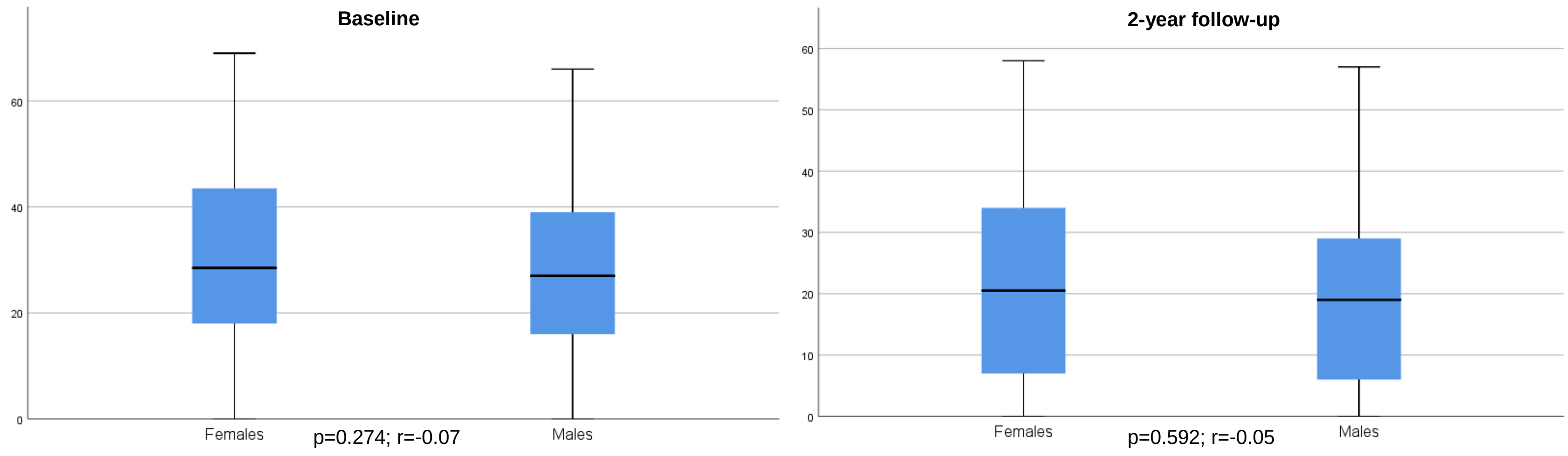

Sustained attention (Continuous Performance Test–II, CPT-II)

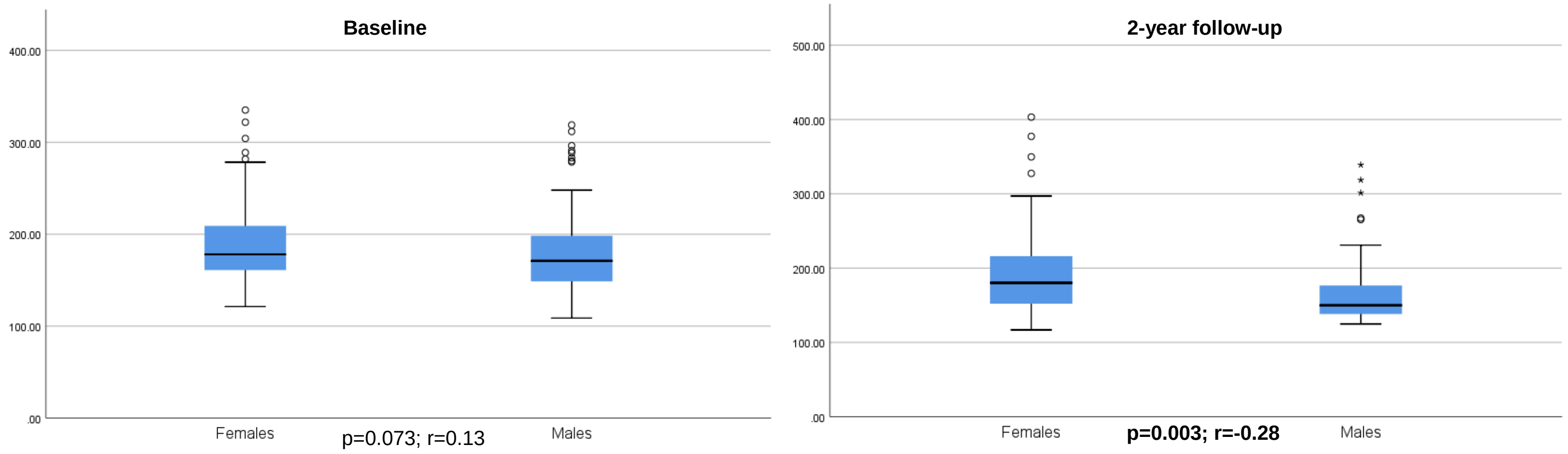

Verbal Memory (Verbal Learning Test Spain Complutense for adults, TAVEC)

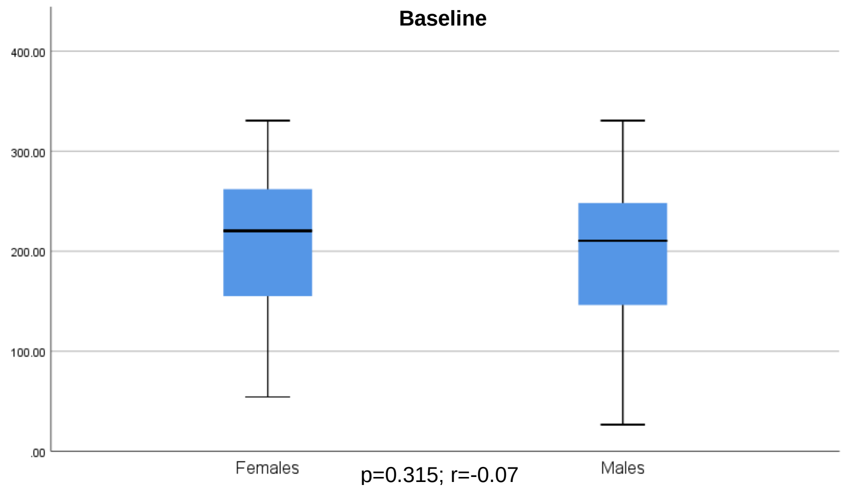

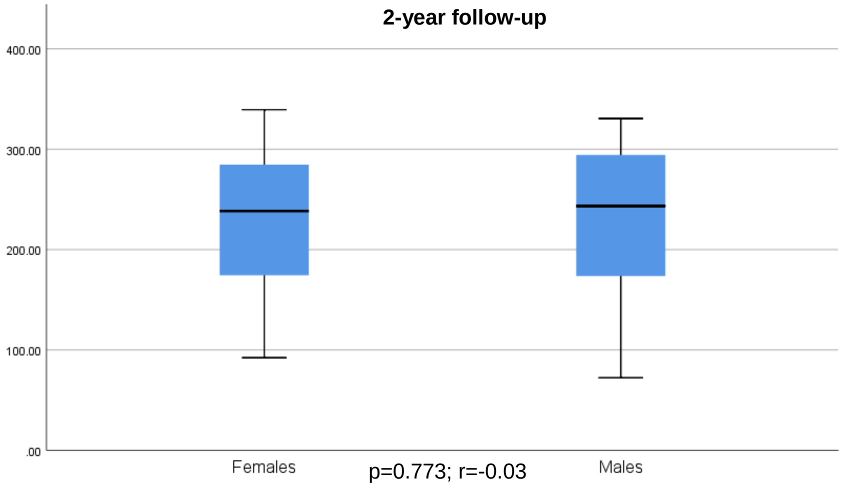

Working Memory (Digit Span Subtest and the Letter-Number Sequencing Subtest of the Wechsler Adult Intelligence Scale)

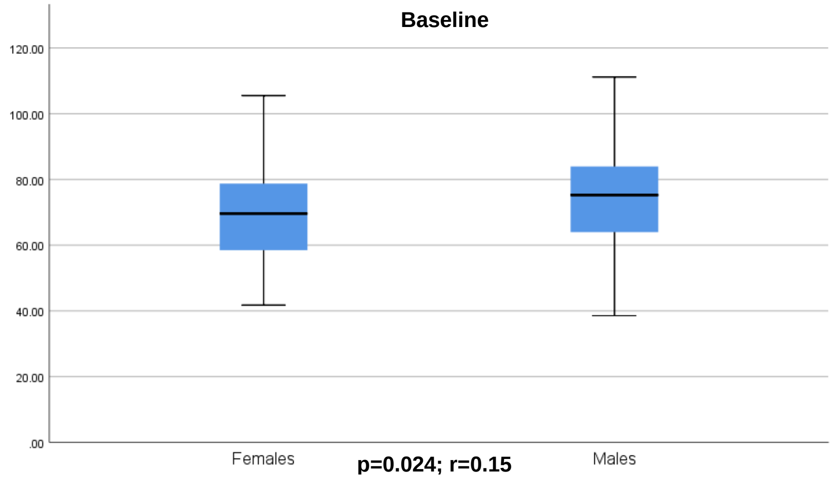

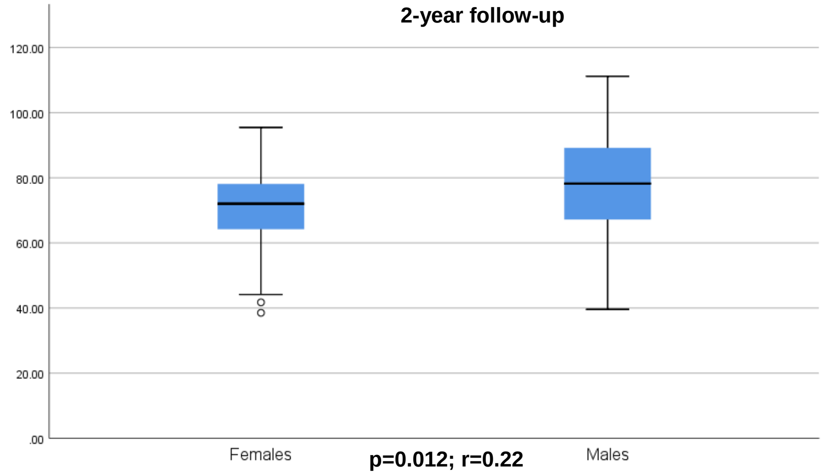

Processing speed (Trail making test (Form A), TMT-A)

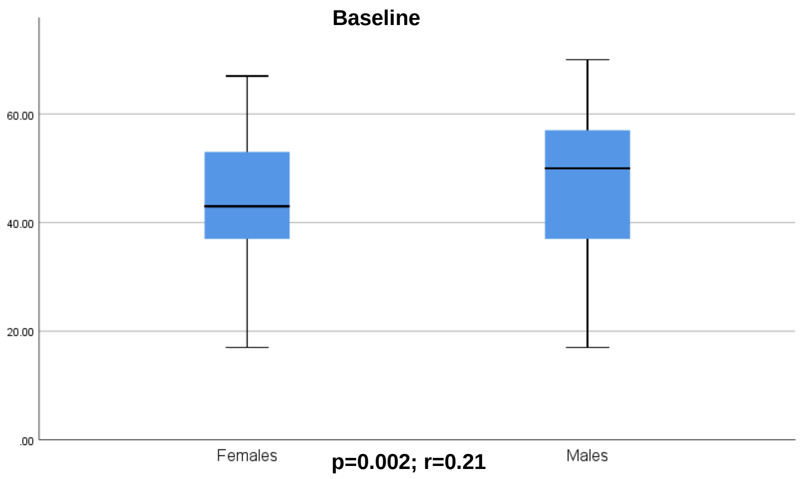

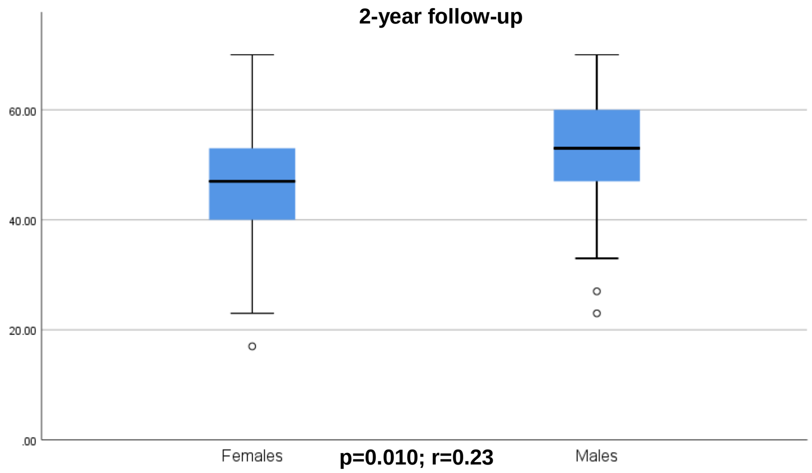

Executive Function (Wisconsin Card Sorting Test)

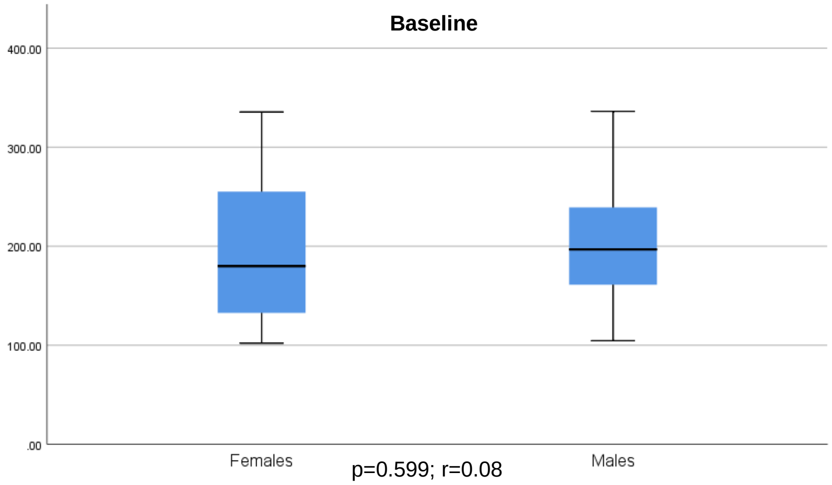

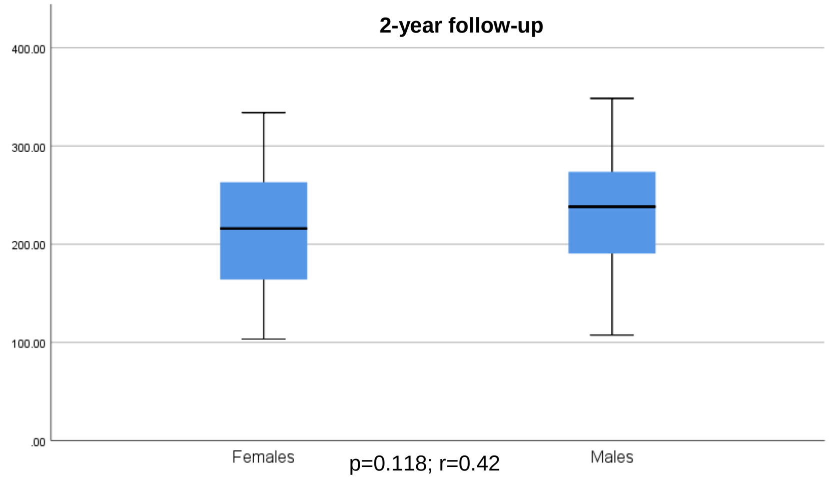

Fluency (semantic fluency (animals) and F-A-S tests)

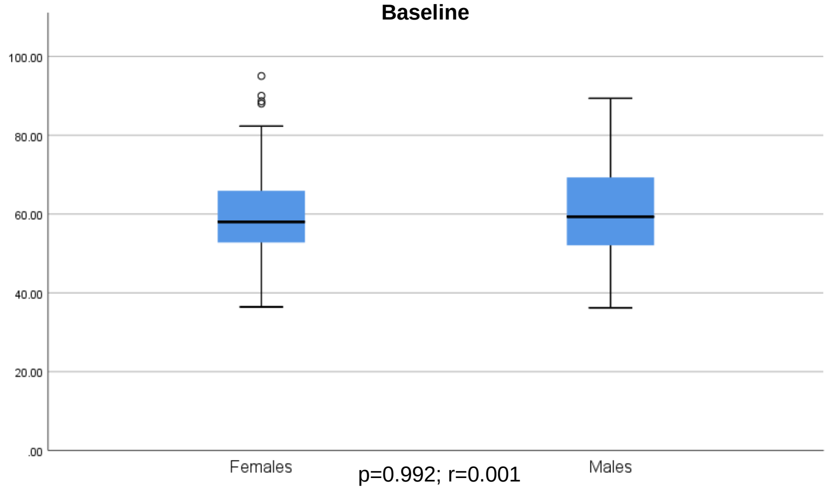

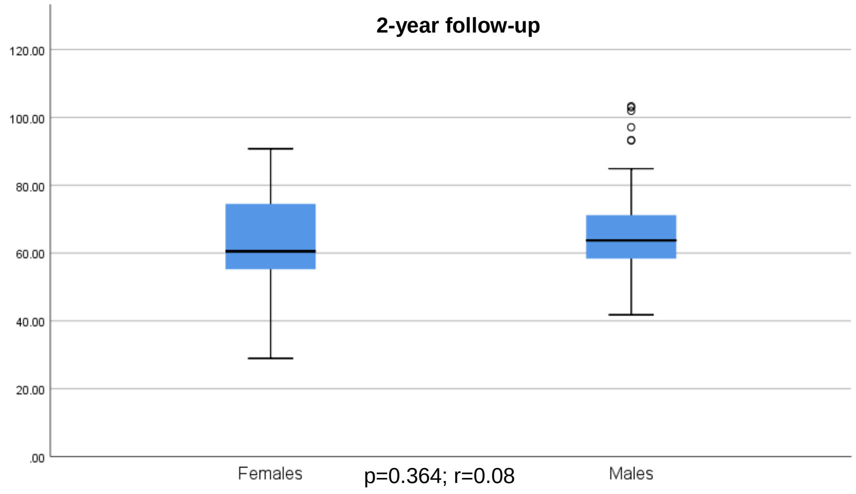

Emotional Intelligence (Mayer-Salovey-Caruso Emotional Intelligence Test, MSCEIT)

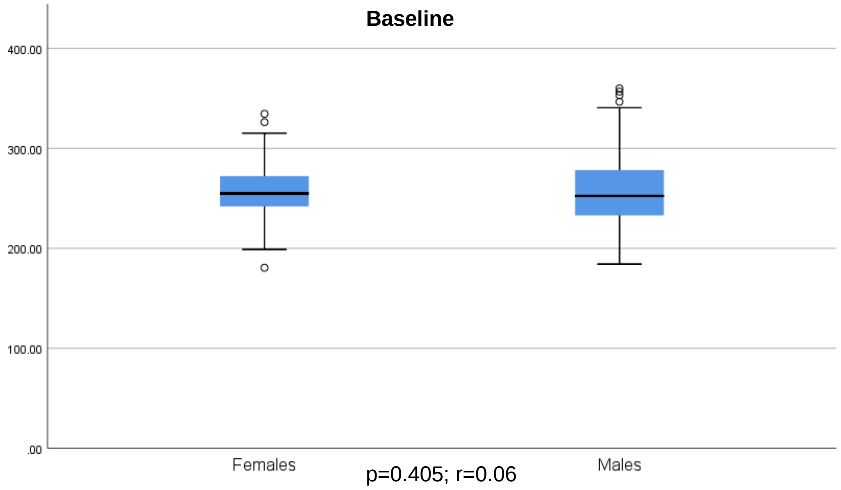

p=0.405; r=0.06

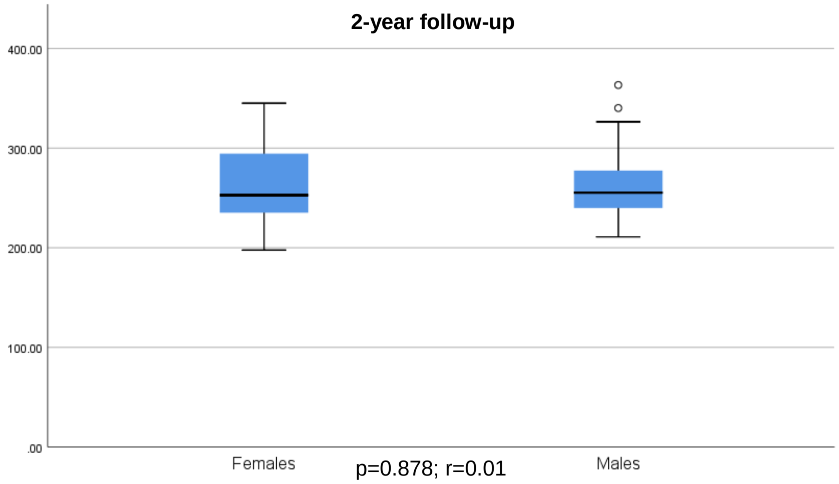

p=0.878; r=0.01
