# Supplementary material for: Influence of clinical and neurocognitive factors in psychosocial functioning after a first episode non-affective psychosis: differences between males and females
Source: Front Psychiatry. 2022 Oct 20;13:982583. doi: 10.3389/fpsyt.2022.982583 (PMC9632657; doi:10.3389/fpsyt.2022.982583)
Supplement: Supplementary file 3 [file Table_1.DOCX]

**Supplementary Table 1.** Principal Component Analysis (PCA) for cognitive variables.

| **Cognitive domain** | **Cognitive test** | **Pattern coefficients** | | | | | **Structure coefficients** | | | | | **Communalities** |
| --- | --- | --- | --- | --- | --- | --- | --- | --- | --- | --- | --- | --- |
|  |  | **1** | **2** | **3** | **4** | **5** | **1** | **2** | **3** | **4** | **5** |  |
| **Verbal memory** | Long Delay Free Recall (CVLT) | **0.945** | 0.021 | 0.028 | 0.001 | -0.022 | **0.945** | 0.217 | -0.368 | 0.342 | 0.256 | 0.875 |
|  | Short Delay Free Recall (CVLT) | **0.938** | 0.008 | 0.057 | -0.048 | 0.002 | **0.939** | 0.253 | -0.325 | 0.340 | 0.289 | 0.819 |
|  | Long Delay Cued Recall (CVLT) | **0.938** | -0.009 | -0.046 | -0.022 | 0.005 | **0.935** | 0.237 | -0.307 | 0.345 | 0.230 | 0.894 |
|  | Short Delay Cued Recall (CVLT) | **0.928** | 0.035 | 0.007 | -0.018 | 0.042 | **0.909** | 0.191 | -0.324 | 0.324 | 0.266 | 0.884 |
|  | Total trial 5 (CVLT) | **0.911** | -0.025 | -0.011 | -0.020 | 0.026 | **0.902** | 0.210 | -0.260 | 0.286 | 0.239 | 0.827 |
|  | Total trials 1–5 (CVLT) | **0.827** | -0.004 | -0.041 | 0.066 | 0.058 | **0.880** | 0.215 | -0.357 | 0.396 | 0.295 | 0.785 |
| **Executive function** | Total errors (WCST) | 0.001 | **0.973** | -0.017 | 0.012 | -0.008 | 0.240 | **0.978** | -0.203 | 0.189 | 0.076 | 0.957 |
|  | Perseverative error (WCST) | -0.084 | **0.938** | -0.006 | -0.050 | 0.037 | 0.239 | **0.933** | -0.194 | 0.202 | 0.066 | 0.845 |
|  | Conceptual response (WCST) | 0.010 | **0.924** | -0.008 | 0.036 | -0.020 | 0.131 | **0.913** | -0.140 | 0.093 | 0.083 | 0.872 |
|  | Perseverative responses (WCST) | 0.011 | **0.912** | 0.026 | -0.081 | 0.062 | 0.204 | **0.901** | -0.130 | 0.088 | 0.122 | 0.821 |
|  | No perseverative responses (WCST) | 0.077 | **0.785** | 0.013 | 0.089 | -0.081 | 0.269 | **0.810** | -0.180 | 0.238 | 0.018 | 0.673 |
| **Sustained attention** | Variability (CPT-II) | 0.036 | -0.003 | **0.933** | 0.065 | -0.135 | -0.305 | -0.169 | **0.914** | -0.234 | -0.209 | 0.855 |
|  | Hit RT Std. Error (CPT-II) | 0.069 | -0.033 | **0.925** | 0.084 | -0.153 | -0.274 | -0.188 | **0.897** | -0.209 | -0.216 | 0.832 |
|  | Perseverative response (CPT-II) | -0.129 | 0.021 | **0.654** | -0.069 | 0.061 | -0.364 | -0.139 | **0.711** | -0.305 | -0.050 | 0.527 |
|  | Omissions (CPT-II) | -0.094 | -0.038 | **0.563** | -0.187 | 0.383 | -0.269 | -0.168 | **0.623** | -0.336 | 0.265 | 0.547 |
| **Working memory** | Letter-number (WAIS-III) | 0.009 | -0.020 | 0.078 | **0.899** | -0.026 | 0.306 | 0.125 | -0.197 | **0.870** | 0.124 | 0.764 |
|  | Digits (WAIS-III) | -0.050 | 0.047 | -0.058 | **0.766** | 0.180 | 0.316 | 0.196 | -0.304 | **0.805** | 0.310 | 0.683 |
| **Verbal fluency** | Categorical (Animal naming) | 0.145 | -0.015 | -0.087 | 0.169 | **0.720** | 0.428 | 0.124 | -0.260 | 0.373 | **0.796** | 0.719 |
|  | Phonemic (F‐A‐S) | 0.191 | 0.080 | -0.129 | 0.154 | **0.658** | 0.489 | 0.230 | -0.326 | 0.394 | **0.756** | 0.712 |

The 19 items were subjected to principal components analysis (PCA) using SPSS version 25. The Kaiser-Meyer-Olkin value was 0.878. exceeding the recommended value of 0.6 and Bartlett’s Test of Sphericity reached statistical significance. supporting the factorability of the correlation matrix. Principal components analysis revealed the presence of five components with eigenvalues exceeding 1. explaining 38.15%. 18.35%. 10.46%. 6.78% and 4.64% of the variance respectively.

Abbreviations: CVLT=California Verbal Learning Test; WCST=Wisconsin Card Sorting Test; CPT-II=Continuous Performance Test–II; WAIS-III=Weschler Adult Intelligence Scale. Third Edition.
