# Supplementary material for: Influence of clinical and neurocognitive factors in psychosocial functioning after a first episode non-affective psychosis: differences between males and females
Source: Front Psychiatry. 2022 Oct 20;13:982583. doi: 10.3389/fpsyt.2022.982583 (PMC9632657; doi:10.3389/fpsyt.2022.982583)
Supplement: Supplementary file 4 [file Table_2.DOCX]

**Supplementary Table 2.** Sex differences in sociodemographic, clinical and functional characteristics at follow-up for subjects with psychosis and healthy controls

|  | **Subjects with psychosis (n=145)** | | **Healthy controls (n=152)** | | **Effect** | | | | | | | | | |  |
| --- | --- | --- | --- | --- | --- | --- | --- | --- | --- | --- | --- | --- | --- | --- | --- |
|  | **Males (n=97)** | **Females (n=48)** | **Males (n=101)** | **Females (n=51)** | **Group** | | | **Sex** | | | **GroupXSex** | | | |  |
|  |  |  |  |  | **F or χ2** | **p** | **ηp ^2^** | **F or χ2** | **p** | **ηp ^2^** | **F or χ2** | **p** | **ηp ^2^** | |  |
| **Sociodemographic variables** | | | | | | | | | | | | | | | |
| Tobacco use: Yes *N* (%) | 60 (37) | 27 (31) | 38 (27) | 17 (21) | 18.714 | **<0.001** | 0.199 | 2.812 | 0.245 | 0.077 |  |  |  | |  |
| Cannabis use: Yes *N* (%) | 16 (10) | 10 (12) | 31 (22) | 4 (5) | 4.210 | 0.122 | 0.095 | 5.597 | 0.061 | 0.109 |  |  |  | |  |
| **Clinical and functional variables** (M±SD) | | | | | | | | | | | | | | | |
| PANSS positive | 10.41±3.89 | 10.69±5.72 | - | - | - | - | - | 0.121 | 0.729 | 0.001 | - | - | - | |  |
| PANSS negative | 14.74±6.53 | 13.90±6.24 | - | - | - | - | - | 0.550 | 0.459 | 0.004 | - | - | - | |  |
| PANSS general | 25.89±8.93 | 26.42±8.95 | - | - | - | - | - | 0.113 | 0.737 | 0.001 | - | - | - | |  |
| PANSS total | 51.03±17.47 | 51.00±19.19 | - | - | - | - | - | 0.000 | 0.992 | 0.000 | - | - | - | |  |
| MADRS score | 5.89±6.28 | 7.43±7.69 | - | - | - | - | - | 1.636 | 0.203 | 0.011 | - | - | - | |  |
| YMRS score | 1.75±3.06 | 3.27±6.53 | - | - | - | - | - | 3.645 | 0.058 | 0.025 | - | - | - | |  |
| CPZ | 59.23±178.03 | 68.96±193.75 | - | - | - | - | - | 0.323 | 0.570 | 0.001 | - | - | - | |  |
| FAST | 19.55±14.73 | 20.98±15.23 | 2.68±7.49 | 3.30±10.00 | 132.792 | **<0.001** | 0.316 | 0.470 | 0.494 | 0.002 | 0.073 | 0.788 | 0.000 | |  |
| **Cognitive measures** (M±SD) | | | | | | | | | | | | | |  | |
| Attention | 166.47±43.98 | 199.59±70.54 | 140.08±19.64 | 148.06±21.20 | 6.202 | **0.013** | 0.025 | 24.176 | **<0.001** | 0.092 | 10.028 | **0.002** | 0.040 | |  |
| Verbal memory | 229.68±71.64 | 233.51±66.14 | 306.72±32.23 | 309.01±37.21 | 37.941 | **<0.001** | 0.130 | 0.023 | 0.879 | 0.000 | 0.169 | 0.681 | 0.001 | |  |
| Working memory | 78.26±14.90 | 71.19±14.34 | 95.41±35.33 | 92.59±47.87 | 7.148 | **0.008** | 0.027 | 2.727 | 0.100 | 0.010 | 0.669 | 0.414 | 0.003 | |  |
| Processing speed | 52.55±10.40 | 47.26±11.41 | 60.37±8.28 | 58.67±7.15 | 31.512 | **<0.001** | 0.109 | 8.402 | **0.004** | 0.032 | 2.742 | 0.099 | 0.011 | |  |
| Executive function | 236.01±55.74 | 218.26±63.51 | 251.07±42.57 | 245.78±41.66 | 4.761 | **0.030** | 0.019 | 2.847 | 0.093 | 0.012 | 1.329 | 0.250 | 0.005 | |  |
| Fluency | 65.88±13.48 | 63.49±13.88 | 79.08±11.37 | 81.12±14.23 | 36.069 | **<0.001** | 0.127 | 0.133 | 0.716 | 0.001 | 2.120 | 0.147 | 0.008 | |  |
| Managing Emotions | 261.72±30.85 | 260.73±34.42 | 286.38±32.41 | 287.67±31.20 | 19.338 | **<0.001** | 0.077 | 0.013 | 0.911 | 0.000 | 0.150 | 0.699 | 0.001 | |  |

Abbreviations: M=Mean; PANSS= Positive and Negative Symptom Scale; MADRS= Montgomery-Asberg Depression Rating Scale; YMRS= Young Mania Rating Scale; DUP= Duration of Untreated Psychosis; CPZ= Chlorpromazine equivalents; FAST=Functioning Assessment Short Test. Significant differences (p<0.05) marked in bold.
