# Supplementary material for: Influence of clinical and neurocognitive factors in psychosocial functioning after a first episode non-affective psychosis: differences between males and females
Source: Front Psychiatry. 2022 Oct 20;13:982583. doi: 10.3389/fpsyt.2022.982583 (PMC9632657; doi:10.3389/fpsyt.2022.982583)
Supplement: Supplementary file 5 [file Table_3.DOCX]

**Supplementary Table 3.** Proportion (percentage) of sample showing significant psychosocial functioning and cognitive changes using the reliable change index (RCI) method

|  | **Males** | | | **Females** | | | **χ2** | **p** | **Cramér's V** |
| --- | --- | --- | --- | --- | --- | --- | --- | --- | --- |
|  | **Unchanged** | **Deteriorated** | **Improved** | **Unchanged** | **Deteriorated** | **Improved** |  |  |  |
| FAST | 6.1 | 26.5 | 67.4 | 6.5 | 26.1 | 67.4 | 0.010 | 0.995 | 0.008 |
| Attention | 16.2 | 42.6 | 41.2 | 8.8 | 35.3 | 55.9 | 2.262 | 0.323 | 0.149 |
| Verbal memory | 13.0 | 41.5 | 45.5 | 17.9 | 38.5 | 43.6 | 0.516 | 0.773 | 0.067 |
| Working memory | 46.8 | 20.3 | 32.9 | 88.1 | 4.8 | 7.1 | 19.654 | **<0.001** | 0.403 |
| Processing speed | 11.2 | 43.8 | 45.0 | 2.4 | 54.8 | 42.8 | 3.374 | 0.185 | 0.166 |
| Executive function | 8.0 | 60.0 | 32.0 | 17.5 | 42.5 | 40.0 | 4.045 | 0.132 | 0.188 |
| Fluency | 17.8 | 52.1 | 30.1 | 28.9 | 31.6 | 39.5 | 4.414 | 0.110 | 0.199 |
| Managing Emotions | 25.0 | 48.2 | 26.8 | 21.2 | 48.5 | 30.3 | 0.218 | 0.897 | 0.049 |

Abbreviations: FAST=Functioning Assessment Short Test. Significant differences (p<0.05) marked in bold.
