# Supplementary material for: Influence of clinical and neurocognitive factors in psychosocial functioning after a first episode non-affective psychosis: differences between males and females
Source: Front Psychiatry. 2022 Oct 20;13:982583. doi: 10.3389/fpsyt.2022.982583 (PMC9632657; doi:10.3389/fpsyt.2022.982583)
Supplement: Supplementary file 6 [file Table_4.DOCX]

**Supplementary Table 4.** Correlations between psychosocial functioning (FAST) and socio-demographic and clinical variables at baseline in subjects with psychosis and healthy controls

|  | **Subjects with psychosis (n=247)** | | | | **Healthy controls (n=224)** | | | |
| --- | --- | --- | --- | --- | --- | --- | --- | --- |
|  | **Males (n=161)** | | **Females (n=86)** | | **Males (n=142)** | | **Females (n=82)** | |
|  | **Pearson**  **Correlation, Student t, X^2^** | **p** | **Pearson**  **Correlation, Student t, X^2^** | **p** | **Pearson**  **Correlation, Student t, X^2^** | **p** | **Pearson**  **Correlation, Student t, X^2^** | **p** |
| **Sociodemographic variables** | | | | | | | | |
| Age (M±SD) | -0.003 | 0.970 | -0.023 | 0.840 | -0.133 | 0.120 | 0.017 | 0.881 |
| SES (%) | 2.062 | 0.073 | 2.160 | 0.067 | 0.201 | 0.962 | 7.742 | **<0.001** |
| Tobacco use: Yes *N* (%) | 1.519 | 0.391 | 0.391 | 0.697 | 1.307 | 0.194 | 0.206 | 0.837 |
| Cannabis use: Yes *N* (%) | 0.579 | 0.564 | 0.740 | 0.461 | 0.415 | 0.679 | 1.112 | 0.270 |
| **Clinical and functional variables** (M±SD) | | | | | | | | |
| PANSS positive | 0.308 | **<0.001** | 0.467 | **<0.001** | - | - | - | - |
| PANSS negative | 0.234 | **0.004** | 0.613 | **<0.001** | - | - | - | - |
| PANSS general | 0.317 | **<0.001** | 0.567 | **<0.001** | - | - | - | - |
| PANSS total | 0.364 | **<0.001** | 0.650 | **<0.001** | - | - | - | - |
| MADRS score | 0.287 | **<0.001** | 0.358 | **0.001** | - | - | - | - |
| YMRS score | 0.231 | **0.005** | 0.272 | **0.017** | - | - | - | - |
| DUP | 0.125 | 0.157 | 0.018 | 0.882 | - | - | - | - |
| **Cognitive measures** (M±SD) | | | | | | | | |
| Attention | 0.112 | 0.242 | 0.299 | **0.025** | 0.027 | 0.768 | -0.103 | 0.401 |
| Verbal memory | -0.130 | 0.143 | -0.118 | 0.358 | -0.030 | 0.735 | -0.345 | **0.003** |
| Working memory | -0.168 | 0.052 | -0.109 | 0.389 | -0.138 | 0.114 | -0.085 | 0.468 |
| Processing speed | -0.183 | **0.035** | -0.055 | 0.659 | -0.060 | 0.490 | -0.152 | 0.192 |
| Executive function | 0.048 | 0.593 | 0.006 | 0.963 | 0.014 | 0.883 | -0.076 | 0.534 |
| Fluency | -0.042 | 0.639 | -0.218 | 0.089 | -0.155 | 0.078 | -0.281 | **0.018** |
| Managing Emotions | -0.213 | **0.017** | -0.181 | 0.156 | -0.027 | 0.756 | -0.227 | 0.057 |
| **Cognitive reserve and premorbid adjustment** (M±SD) | | | | | | | | |
| CR | -0.083 | 0.334 | -0.112 | 0.369 | 0.062 | 0.479 | -0.217 | 0.063 |
| PAS | 0.228 | **0.006** | 0.433 | **<0.001** | 0.324 | **<0.001** | 0.426 | **<0.001** |

Abbreviations: M=Mean; SES=Socioeconomic status; PANSS= Positive and Negative Symptom Scale; MADRS= Montgomery-Asberg Depression Rating Scale; YMRS= Young Mania Rating Scale; DUP= Duration of Untreated Psychosis; CPZ= Chlorpromazine equivalents; FAST=Functioning Assessment Short Test; CR= Cognitive Reserve, PAS= Premorbid Adjustment Scale. Significant differences (p<0.05) marked in bold.
